# Genomic Signatures of Local Adaptation in Clam Shrimp (*Eulimnadia texana*) from Natural Vernal Pools

**DOI:** 10.1093/gbe/evaa120

**Published:** 2020-06-15

**Authors:** James G Baldwin-Brown, Anthony D Long

**Affiliations:** e1 School of Biological Sciences, University of Utah; e2 Department of Ecology and Evolutionary Biology, University of California Irvine

**Keywords:** adaptation, ecological genetics, genomics/proteomics, landscape genetics, natural selection and contemporary evolution, population genetics: empirical

## Abstract

Vernal pools are unique in their isolation and the strong selection acting on their resident species. Vernal pool clam shrimp (*Eulimnadia texana*) are a promising model due to ease of culturing, short generation time, small genomes, and obligate desiccated diapaused eggs. Clam shrimp are also androdioecious (sexes include males and hermaphrodites), and here we use population-scaled recombination rates to support the hypothesis that the heterogametic sex is recombination free in these shrimp. We collected short-read sequence data from pooled samples from different vernal pools to gain insights into local adaptation. We identify genomic regions in which some populations have allele frequencies that differ significantly from the metapopulation. *BayPass* (Gautier M. 2015. Genome-wide scan for adaptive divergence and association with population-specific covariates. Genetics 201(4):1555–1579.) detected 19 such genomic regions showing an excess of population subdivision. These regions on average are 550 bp in size and had 2.5 genes within 5 kb of them. Genes located near these regions are involved in Malpighian tubule function and osmoregulation, an essential function in vernal pools. It is likely that salinity profiles vary between pools and over time, and variants at these genes are adapted to local salinity conditions.

## Introduction

The clam shrimp *Eulimnadia texana* is known for its unique sex-determining system (Sassaman and Weeks 1993), its rare (in Metazoa) requirement to reproduce via desiccated diapaused eggs (Sassaman and Weeks 1993), and its unique habitat. *Eulimnadia texana* is a rare androdioecious (Sassaman and Weeks 1993) species with three common arrangements of “proto-sex chromosomes” (Sassaman and Weeks 1993; Weeks et al. 2010). The ability of eggs to remain in diapause for years at a time (Brendonck 1996) is especially valuable to geneticists because very few macroscopic animals exist for which populations can be archived for long periods without change in allele frequency and linkage disequilibrium (LD) occurring. In a previous article, we carried out a highly contiguous genome assembly of *E. texana* with a contig N50 of 18 Mb and a genome only 120 Mb in total size (Baldwin-Brown et al. 2018). *Eulimnadia texana* shrimp live in isolated vernal pools in the desert southwest of the United States. Prior studies (Bohonak 1998) indicate naturally limited migration from pool to pool, making *E. texana* well suited to the study of populations evolving in relative genetic isolation, although the data of this work suggest that migration rates are higher than previously assumed.

Various methods (Weir and Cockerham 1984; Nielsen et al. 2005; Voight et al. 2006; Frichot et al. 2013; Günther and Coop 2013) have been proposed for identifying molecular markers important in local adaptation between populations. A high *F*_ST_ value at some locus relative to the genome-wide background indicates that a force outside genetic drift and migration is acting upon variation at that locus (Akey et al. 2002). The *X^T^X* statistic (Goodman 1999; Günther and Coop 2013) employed by *BayPass* (Gautier 2015) is analogous to *F*_ST_ in that both test for differentiation between populations larger than expected by chance in a manner agnostic to environment covariates. *BayPass*-related Bayes factor statistic can further suggest environmental variables that are important drivers of adaptation by identifying covariation between allele frequency and environmental covariates. These newer approaches are believed to be more powerful than traditional approaches based on *F*_ST_ (Lotterhos and Whitlock 2014).

Pooled population sequencing (Poolseq), where multiple individuals from a population are pooled and short-read sequenced, allows for the inexpensive estimation of allele frequencies for multiple populations (Burke et al. 2010; Futschik and Schlötterer 2010). We used Poolseq to obtain such genome-wide allele frequency estimate for several shrimp populations. We used these data to identify single-nucleotide polymorphisms (SNPs) and estimate classical population genetics parameters, such as Watterson’s theta (*θ*) (Watterson 1975) and the population-adjusted recombination rate, rho (*ρ*) (Stumpf and McVean 2003). We further estimate both *F*_ST_ (Hivert et al. 2018) and the *X^T^X* statistic using *BayPass*, then use *BayPass*’ Bayes factor statistic to identify genome regions potentially under local adaptation. Both *F*_ST_ and *BayPass* gave qualitatively similar results. *BayPass* identifies 19 regions showing significant population differentiation. We argue that the regions identified by *BayPass* are more likely to be true positives than those identified via traditional *F*_ST_-based approaches.

The Poolseq data are suitable for addressing other questions related to the genetics and biology of the clam shrimp. *Eulimnadia texana* has a unique sex determination system in which an individual can be male or hermaphroditic, with males having the ZZ genotype and hermaphrodites being either amphigenic (ZW) or monogenic (WW) genotypes. The Z and W alleles are transmitted according to Mendelian ratios, and only the amphigenic ZW-carrying hermaphrodites are heterogametic. Numerous organisms do not recombine chromosomes in the heterogametic sex, including *Drosophila melanogaster*, but little is known about heterogametic recombination in amphigenic animals, especially those that, like *E. texana*, have recently diverged sex chromosomes. Some evidence exists that heterogametic *E. texana* do not recombine (Baldwin-Brown et al. 2018). We show that short-range population-based estimates of recombination rate are consistent with loss of recombination in heterogametic hermaphrodites.

## Materials and Methods

### Shrimp Collection and Rearing

**Fig. 1.**
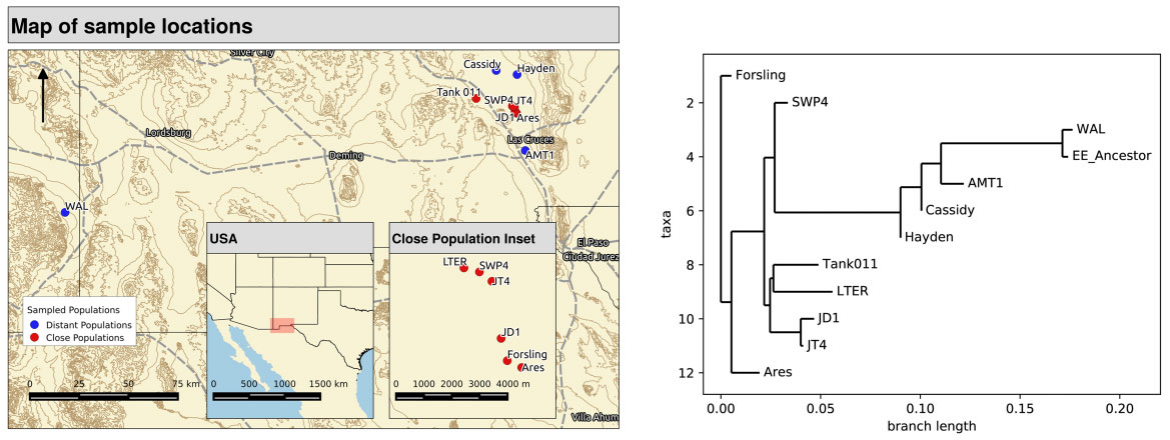
A map of the sampling locations for the 11 study populations and a maximum likelihood tree generated by TreeMix depicting the relatedness of the populations based on genome-wide allele frequency estimates. All populations were taken as soil samples from field sites in New Mexico and Arizona. The “EE_Ancestor” strain is a laboratory strain descended from WAL.

Clam shrimp populations were sampled from New Mexico and Arizona. Samples were collected in six separate summers (1995, 1996, 1998, 2000, 2003, and 2004). Ecological measurements were made on dry pools, and dry soil samples were collected and hydrated in the laboratory to produce shrimp for study. Although these samples are not very evenly distributed geographically, they have the advantage of having previously been used for estimation of inbreeding, and details on collection and rearing are published (Weeks and Zucker 1999). We acquired 11 soil samples, each from a different natural vernal pool, to grow clam shrimp for sequencing (fig. 1 and supplementary tables 1 and 2, Supplementary Material online). We also sequenced one laboratory population (EE) that was derived from 265 WAL wild individuals carried through six laboratory generations with a minimum population size of 250. Populations were reared in 50X30X8 cm aluminum foil catering trays (Catering Essentials, full size steam table pan). In each tray, we mixed 500 ml of soil with 6 l of water purified via reverse osmosis; 0.3 g of Aquarium salt (API aquarium salt, Mars Fishcare North America, Inc.) was added to each tray to ensure that necessary nutrients were available to the shrimp. Trays were checked daily for nonclam shrimp, which were immediately removed from trays. We identified the following nonclam shrimp: *Triops longicaudatus*, *Daphnia pulex*, and an unknown species of *Anostraca* fairy shrimp.

### Illumina Library Preparation and Sequencing

We hydrated the soil samples and then collected 100 individuals (males and females) from each population on day 10 of their life cycle. We prepared barcoded (supplementary table 1, Supplementary Material online) gDNA libraries using the Nextera Library Preparation Kit and DNA from the pool of 100 individuals. Thirteen cycles of polymerase chain reaction (PCR) were used during the Nextera protocol, except in the case of the LTER and Tank 011 populations, where 15 cycles of PCR were used to increase yield. Libraries were pooled and size selected on a Pippin (Sage Science, Beverly, MA) size selection instrument. The pooled library was sequenced over four lanes of paired-end 100-bp Illumina sequencing on an Illumina HiSeq 2500 generating a total of 127 Gb of data, or 844× of coverage (supplementary table 2, Supplementary Material online), averaging 70× of coverage per pooled population.

### SNP Calling

We explored four variant calling pipelines before choosing the one described here (see Supplementary Material online for a comparison of pipelines). Our chosen pipeline consisted of calling SNPs using *GATK*’s (McKenna et al. 2010) *HaplotypeCaller* with the default (diploid) settings. We filtered on a minimum quality of 30 and removed all SNPs with mapped coverages below 10 in any population. Command line options for *Picard tools*, *BWA* (Li and Durbin 2009), and *GATK* are included in the supplementary texts, Supplementary Material online, and the full scripts are available at GitHub. This pipeline was not able to detect very rare polymorphic sites in many cases (see site frequency spectrum, supplementary figs. 1–5, Supplementary Material online). This being said, rare polymorphic sites cannot be easily accommodated using the *BayPass* software and are unlikely to show allele frequency differences between populations at any rate.

### Identification of Candidate Genomic Regions, Including Using Correlations with Environmental Variables

We used *F*_ST_ and *BayPass*’ *X^T^X* to identify population differentiation in the sequenced populations. *F*_ST_ is a classical population genetic statistic that may be interpreted as the fraction of allele frequency variance due to differences among populations. *X^T^X* is a Bayesian measure of the deviation of subpopulations’ allele frequencies from their expected frequencies due to shared ancestry in the populations and is calculated via a Markov chain Monte Carlo approximation. *X^T^X* is high when the allele frequency differences between populations are higher than expected at a site. In addition to *X^T^X*, *BayPass*’ Bayes factors identify population differentiation associated with ecological variables measured for each population. Bayes factors are similar to *X^T^X* in that they are a Bayesian measure of allele frequency deviation from expectations due to shared ancestry but differ in that they identify deviations that correlate with an environmental variable measured on each population. The Bayes factor is a ratio of the likelihood of a model including both ancestry and an environmental variable divided by the likelihood of a model based on ancestry alone.

All of the environmental variables that we associated with allele frequency differences are derived from measurements taken in the field during sample collection. Some environmental variables require special description. “Date” is the date of collection of the soil. “Percent males” refers to the fraction of individuals that were male in hydrated samples. The proportion of males is a proxy for the level of self-fertilization in that population. A fully self-fertilizing population will be 0% male, whereas a fully outcrossing population will be 50% male, with other self-fertilization proportions linearly related to male proportion. Surface area and volume are calculated based on measurements taken on-site at each vernal pool during the dry season. *Streptocephalus mackeni* and *Thamnocephalus platyurus* refer to the presence of these species of *Anostraca* fairy shrimp, and “Fairy shrimp” refers to fairy shrimp whose species was unknown.

*BayPass* was designed to operate on either separately sequenced individual data or pooled data. In the single-individual-sequencing case, each counted allele represents one of two alleles from a sequenced individual, so a heterozygote would contribute one allele to each of the reference and alternate counts. In the pooled sequencing case (used here), each counted allele represents a single polymorphism call from a single sequencing read, and *BayPass* correctly accounts for the fact that some sequencing reads will represent sequencing from the same individual multiple times.

*BayPass* requires a set of putative neutral sites to calculate the “omega” population distance matrix, which indicates the degree of relatedness between populations. We used 4-fold degenerate sites for this purpose. We generated a custom Python script for identifying 4-fold degenerate sites based on the annotation information produced by *Augustus* (Stanke and Waack 2003) for the reference genome. Candidate sites are only considered 4-fold degenerate if they are 4-fold degenerate for all transcripts overlapping the site. Fourfold degenerate sites are under selection less often than any other class of genomic site (Yang and Bielawski 2000), making them ideal for *BayPass*’s null (omega) covariance matrix estimation. We performed an additional run of *BayPass* covariance matrix generation using only 4-fold degenerate sites separated by at least 100 kb to alleviate concerns about LD influencing covariance matrix estimation but found no substantial differences in the matrices (the correlation between matrices was 0.9995), so the original non-LD-adjusted calculations were used throughout.

We used *poolfstat* (Hivert et al. 2018) to calculate genome-wide *F*_ST_ from the pooled samples, and *npstat* (Ferretti et al. 2013) with a 10-kb window size to calculate Watterson’s *θ*. We used *LDx* to calculate average LD at distances up to 400 bp, and then calculated population-adjusted recombination rate (*ρ*) based on decay of LD (Marroni et al. 2011). To calculate isolation by distance, we performed a linear regression on population pair relating geographic distance to *F*_ST_, produced using either all of the data or all of the data except the more distant WAL population, with the following R command: lm(formula = “*F*_ST_ ∼ distance”), where distance is the distance between pairs of collection sites in kilometers. In addition, we used the default settings of TreeMix (Pickrell and Pritchard 2012) to build a tree describing the relationships between populations based on allele frequency.

### Identifying Peaks of Differentiation

For the *F*_ST_ and *BayPass* analyses, we used a hidden Markov model to identify sites that appeared to be under selection. Following the approach used by Hofer et al. (2012), we used the R package *HiddenMarkov* to run a hidden Markov model on the *X^T^X* scores produced by *BayPass*, thereby identifying the sections of the genome that fit one of two models, one representing the background polymorphisms and the other representing the differentiated peaks. All transition probabilities were set to 0.001, and the Viterbi algorithm was used to estimate the states of the hidden Markov model. All regions classified as belonging to the second state are referred to as “differentiated regions.”

We tested the effect of reduced marker density by uniformly randomly downsampling the *X^T^X* values in our data set. We then reran our hidden Markov model on the downsampled data to recall regions of differentiation to see how effectively regions could be called with reduced markers.

Regions were tested for high coverage by calculating, on a per-population basis, the average coverage in 550-bp tiled windows across the genome (550 bp was the average size of differentiated regions). We then using python’s quantile function to identify the coverage quantile associated with our Bonferroni-corrected alpha for each population. Finally, we checked if average coverage in a region was above this quantile. We assumed 209 tests (11 populations × 19 regions) and an overall false positive rate of 0.05, for an alpha of 0.0003.

### BLAST Annotation

The original annotation of *E. texana* consists of mutual best hit BLAST against the *D. melanogaster* genome (Baldwin-Brown et al. 2018). In the 19 differentiated regions described below, some predicted genes were not successfully annotated via this strategy. For these unannotated genes, we ran a BLAST search against the NCBI *nr* protein database (NCBI Resource Coordinators 2018) and took the most significant BLAST hit for each gene that had an *e*-value below 1 × 10^−5^ and assigned that putative identity to the gene of interest.

## Results

We generated Poolseq data from our 12 populations (fig. 1), calculated allele frequencies at each SNP, and use the resulting allele frequency estimates for subsequent analyses. We used these data for estimation of classical population genetics parameters, for inference about recombination rates in natural populations, and for identification of genomic regions at which populations are differentiated.

### Migration among Pools

We generated a maximum likelihood tree using *Treemix* (Pickrell and Pritchard 2012) to identify relationships among the populations (fig. 1). The populations EE and WAL, being separated by only six generations of laboratory maintenance, show very little differentiation. Several of the natural populations appear to be as closely related to each other as EE and WAL. This observation contradicts the conventional wisdom that vernal pool clam shrimp populations rarely exchange migrants (Bohonak 1998).

**Fig. 2.**
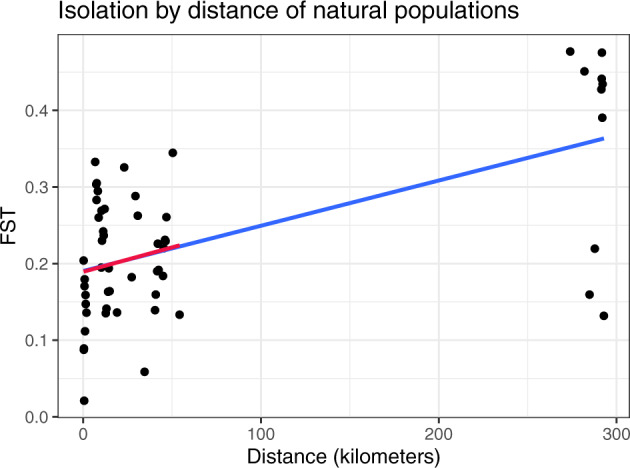
Isolation by distance. This plot depicts the pairwise *F*_ST_ of all natural populations versus the geographical distance between them. The blue line is a linear regression of *F*_ST_ on distance. The red line is the same regression, but excluding the more distant WAL population from the analysis. The slopes of the two regressions are nearly identical.

The inability of vernal pool shrimp to escape the pools in which they are born seems to prohibit migration between distinct pools. A few hundred meters of distance between pools produces genetically distinct populations, as measured by *F*_ST_, in *Anostraca* fairy shrimp (Bohonak 1998). In fact, modest levels of differentiation in allele frequencies between populations (*F*_ST_, as measured by *poolfstat*, is 0.251 across these samples; fig. 1) suggest moderate levels of gene flow between pools. The source of this ability to migrate, whether by animal tracking, wind dispersal, periodic flooding, or some other mechanism, is unknown, but a plot of pairwise *F*_ST_ versus distance is consistent with isolation by distance (fig. 2). The WAL population, being much more distant than other populations, accounts for a large portion of this IBD signal, but the regression line’s slope is minimally changed when WAL is removed (fig. 2).

### The Heterogametic Sex May Not Recombine

We directly measured LD and genome-wide *θ* from our sequencing data, then related those statistics using classical population genetics equations to identify the population-adjusted recombination rate (*ρ*). This demonstrated that the population-wide recombination rate is extremely low in clam shrimp. One plausible explanation for this low recombination rate is lack of recombination in heterogametic individuals.

We used estimated *θ* (Watterson 1975) for Chromosome 1 in the Forsling population using a method that controls for sequencing errors in pooled data (Long et al. 2007) and conditioned on the same set of SNPs as employed in the *BayPass* analyses below. This estimate of *θ* was 0.00347, much lower than the estimate obtained using *npstat* of 0.0156 that uses all SNPs identified via mpileup (Li et al. 2009; Ferretti et al. 2013). We further estimated pi (*π*) using the same set of SNPs as 0.00329 (0.0140 with *npstat*). Under neutrality, *π* and *θ* have the same expectation, so a ratio of *θ*/*π* significantly different from one indicates a departure from neutrality under Wright–Fisher sampling (*D*; Tajima 1989). The *D* calculated from our calls using ascertained SNPs was 1.05, as opposed to 1.12 when using *npstat*’s estimates of *θ* and *π*, suggesting our custom estimate to be slightly more reliable in this case. The expectation of *θ* is 4*N*_e_*μ*, where *μ* is the mutation rate per basepair per gamete per generation. Assuming *μ* here is equal to *Drosophila* at 2.8 × 10^−9^ (Keightley et al. 2014), *N*_e_ = *θ*/4*μ* = 2.97 × 10^5^. We further used *LDx* to calculate average LD at distances up to 400 bp using our same set of SNPs, then calculated recombination rate based on LD decay (Marroni et al. 2011). We estimated the population-adjusted recombination rate (*ρ*) per basepair to be 0.00599. As the expected value of *ρ* is 4*N*_e_*r*, where *r* is the recombination rate per adjacent basepair per gamete per generation, we use the estimate of *N*_e_ obtained above, and our estimated genome size of 120 Mb to estimate a total recombination map for clam shrimp to be 61 cM.

The total number of linkage groups in this species is unknown, but the genome assembly consists of three large contigs and numerous smaller contigs (Baldwin-Brown et al. 2018). Assuming that these large contigs each represent a chromosome and the remaining contigs represent at least one extra chromosome, and assuming an average of either one or two crossovers per chromosome, our a priori expectation for the total map length for *E. texana* is between 200 cM and perhaps 500 cM (if clam shrimp had as many as five total chromosomes). This is in keeping with *D. melanogaster*, which has a similarly sized genome consisting of three chromosomes and a total map length of 279.2 cM (Griffiths et al. 1999). Our total estimated map length of 51 cM is well below one recombination event per chromosome per generation per individual. Some evidence (Baldwin-Brown et al. 2018) indicates that heterogametic (those with ZW sex chromosomes) clam shrimp do not recombine their chromosomes during meiosis. Assuming amphigenic (heterogametic) hermaphrodites are free of recombination, and our estimate of 61 cM is a sex-averaged map length, a typical population consisting of 80% amphigenics (Weeks et al. 1999) would have a male total map length of 305 cM, consistent with the estimated number of chromosomes for this species. These population genetics parameter estimates lend credence to the hypothesis that only monogenic male individuals recombine.

A caveat is that our estimate of *ρ*, calculated from short-range LD, could differ from an LD estimate based on long-range LD (Hill and Weir 1988) and should be updated if long-range LD information becomes available.

### *BayPass* Identifies 19 Narrow Regions Exhibiting Excess Population Differentiation

**Fig. 3.**
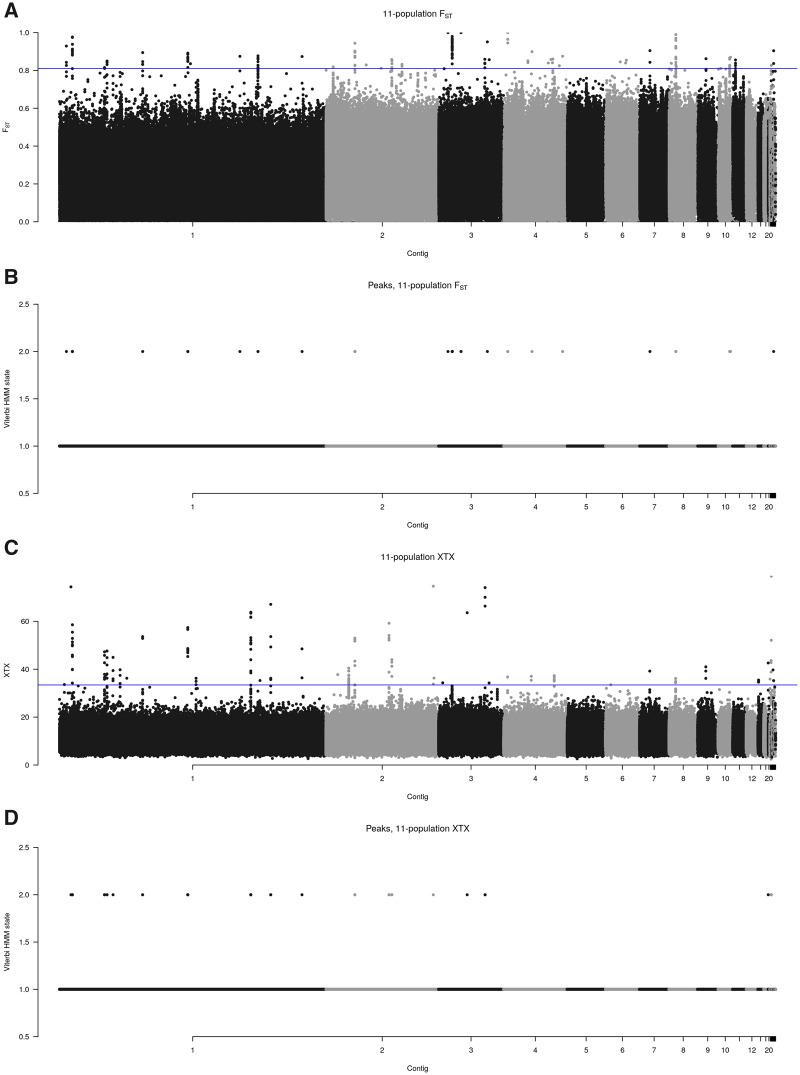
Manhattan plots comparing *F*_ST_ and *X^T^X* (*A* and *C*), demonstrating that peaks of differentiation are identified by both methods but are more clearly resolved using *X^T^X*. Locations of peaks identified by a hidden Markov model tuned to identify outliers of population differentiation (*B* and *D*). Horizontal lines indicate the threshold for the 0.01% most significant loci.

We used *BayPass* and *F*_ST_ to scan for differentiation in all 11 natural populations. We first generated Manhattan plots for *F*_ST_ (fig. 3*A*). A hidden Markov model with two states, background (mean 0.24, standard deviation 0.36) versus differentiated (mean 0.8, standard deviation 1.6), identified 21 differentiation regions (fig. 3*B*). These regions ranged in width from 1 to 2,600 bp and were, on average, 323 bp in width.

**Fig. 4.**
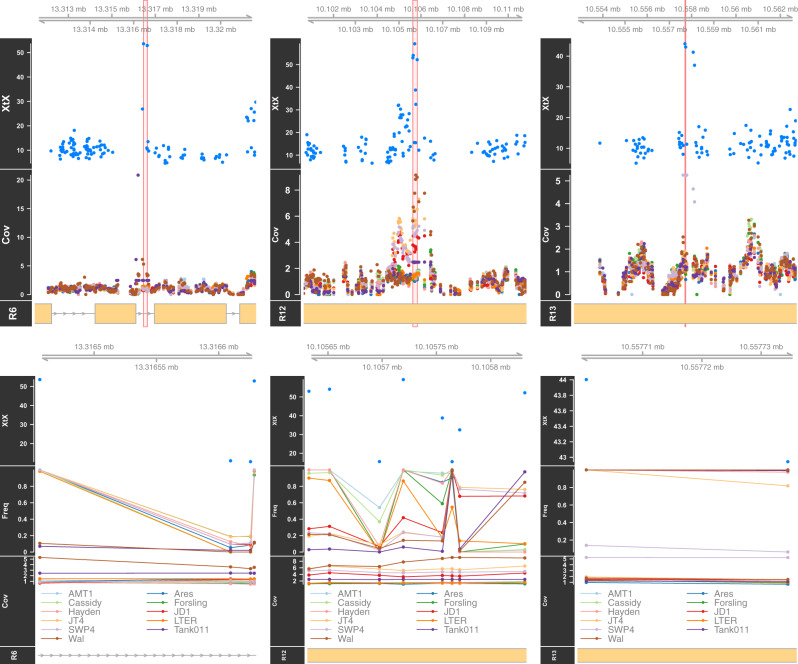
Manhattan plots of single SNP *X^T^X* values indicating excess differentiation among the 11 populations for the regions 6, 12, and 13. The plots indicate that the signal is highly localized, often suggesting a single gene (polycystin-1 for locus 6, Dh44 for locus 12, and nephrin for locus 13). The red rectangle in each plot indicates the region identified as significant by the hidden Markov model. The “R” indicators in the titles indicate the region number. “Freq” refers to the per-population allele frequency at each locus. “Cov” indicates the sequencing depth per population, after normalizing each population-specific coverage to the genome-wide average coverage calculated from 550-bp tiled windows. A coverage of 1 here indicates average coverage, 2 indicates double, etc. The lower plot is a zoomed figure indicating just the region identified as differentiated by the hidden Markov model.

*F*_ST_ is one of the most commonly used statistics for identifying differentiated genomic regions, but it does not correct for the genome-wide relatedness of populations, and it does not take sequencing coverage into account. To remedy both of these problems, we similarly analyzed these data using *BayPass*. Figure 3*C* is a Manhattan plot for *BayPass*’ *X^T^X*. A two-state hidden Markov model (background mean = 20 and SD = 9, differentiated mean = 100 and SD = 200) identified 19 peaks of differentiation across the genome (fig. 3*D*). Regions identified by the hidden Markov model as being in the “differentiated” state within 100 bp one other were combined into a single peak. Five of these peaks (2, 6, 7, 10, and 11) were within 10 kb of peaks identified by *F*_ST_, indicating some agreement between the two methods of differentiated region detection. The peaks identified by the hidden Markov model have two general types of makeup: a subset of SNPs with extremely high *X^T^X* values, scattered throughout a small region (e.g., supplementary fig. 6, regions 2 and 8, Supplementary Material online), or a larger sized region harboring many SNPs with above average *X^T^X* values. The hidden Markov model identified peaks range in width from 1 to 2,600 bp, with an average width of 550 bp (table 1). These peaks consist of one to ∼40 SNPs in regions small enough to contain zero to five gene candidate genes. Figure 4 provides detailed views of the polymorphism data for three regions (6, 12, and 13), chosen because they overlap, respectively, the genes *polycystin-1*, *Dh44*, and *nephrin*, discussed below. These three regions are quite narrow, whereas some others (supplementary fig. 6, Supplementary Material online) are much wider. As the top panels indicate, many of these regions show differences in sequencing coverage between populations that extend beyond the global differences in sequencing coverage, possibly indicating polymorphic repetitive genome features that may influence local adaptation at these sites (fig. 4 and supplementary fig. 6, “Cov” panels, Supplementary Material online). The lower panels indicate that regions 6 and 12 do not have a single outlier population in terms of allele frequency, but in region 13, SWP4 is a single outlier in terms of both allele frequency and sequencing coverage. Both of these patterns are common in other detected regions.

**Table 1:** Major Significant Regions According to the 11-Way *X^T^X* Population Differentiation Analysis

Locus	Contig	Range	Polymorphic Sites in Region	Genes within 5 kb
1	C0001	1,833,709–1,833,710	1	1
2	C0001	2,066,043–2,068,643	43	2
3	C0001	7,209,776–7,210,400	15	3
4	C0001	7,613,579–7,613,897	6	1
5	C0001	8,578,012–8,578,013	1	2
6	C0001	13,316,456–13,316,629	4	4
7	C0001	20,549,353–20,551,632	29	2
8	C0001	30,645,351–30,647,093	31	1
9	C0001	33,839,493–33,840,999	12	2
10	C0001	38,847,716–38,847,717	1	5
11	C0002	4,630,901–4,631,128	7	3
12	C0002	10,105,632–10,105,832	8	1
13	C0002	10,557,700–10,557,735	2	1
14	C0002	17,201,441–17,201,442	1	2
15	C0003	4,598,755–4,598,756	1	2
16	C0003	7,468,748–7,469,069	4	2
17	C0020	27,501–27,502	1	2
18	C0025	29,631–29,634	2	0
19	C0028	32,005–32,415	31	2

Several of the hidden Markov model identified regions are within 5 kb of genes that have well documented functions, including *solute carrier family 35* *member* *E2B* (region 19; hence *E2B*), *ING4* (region 19), *GADPH* (region 1), *bab1* (region 5), *GAT1* (region 6), *polycystin-1* (region 6), *preneuropeptide F II* (region 9), a possible homolog of *OmpA* (region 10), *IFRD1* (region 10), *Cht11* (region 10), *Dh44* (region 12), nephrin-like *isoform* *X2* (region 12), *mucin 12Ea* (region 14), and an *Acetyl-CoA hydrolase/transferase* family protein (region 17). Neuropeptide F influences behavior of *Drosophila* larvae. *Cht11* (*chitinase*) could relate to the strength of the armored shell surrounding the shrimp; similarly, *BAB* is involved in tarsus development in *Drosophila* and *IFRD1* can induce muscular regeneration in humans; all three could be candidates for predator defense genes. Most striking, however, is the strong prevalence of genes relating to osmoregulation and Malpighian tubule and kidney function. *Dh44* (diuretic hormone), *nephrin*, and *polycystin-1* (regions 12, 13, and 6) have functions related to the kidneys or Malpighian tubules, suggesting the importance of osmoregulation or toxin removal to local adaptation. In addition, *GAT1* (region 6) is a sodium- and chloride-dependent GABA transporter. It is conceivable that one avenue for surviving osmotic stress is to adjust ion-dependent transporters to be effective at salinities that match the natural environment of the organism. Osmotic stress is expected to be a common stressor in vernal pools because these pools change volume, and therefore salinity, over time. These genes may differ between populations due to adaptation to local osmotic conditions.

Most of our detected peaks had at least one population in which sequencing coverage was substantially higher than the genome-wide expectation (fig. 4 and supplementary fig. 6, Supplementary Material online). We identified regions with higher-than-expected coverage by calculating, for each population, the genome-wide coverage quantile corresponding to a Bonferroni-corrected percentile (0.9997) for a multi-test-wide alpha of 0.05, then tested whether or not the average population-specific coverage in a region was above that threshold. All 19 regions had at least one population overcovered (fig. 4 and supplementary fig. 6, Supplementary Material online). Only 0.188% of tiled 550 bp regions across the genome had significantly high coverage from at least 1 population, and only 0.00869% of the genome is covered by *X^T^X*-significant regions, indicating that the association between significance and high coverage is not due to chance. Different populations are overrepresented in different peaks, indicating that no one population or sequencing event explains all high-coverage regions. Regions 1–3, 7–12, 17, and 18 are at repeat locations identified by *RepeatMasker* (Chen 2004), whereas regions 4–6, 13–16, and 19 are not. This suggests that regions showing differentiation between populations may often be associated with transposable elements (or other repeats) and/or copy number changes, although additional work would have to be carried out to confirm this suggestion.

### Environmental Variables Sometimes Correlate with Differentiated Regions but Do Not Allow Inference of the Trait under Selection

**Fig. 5.**
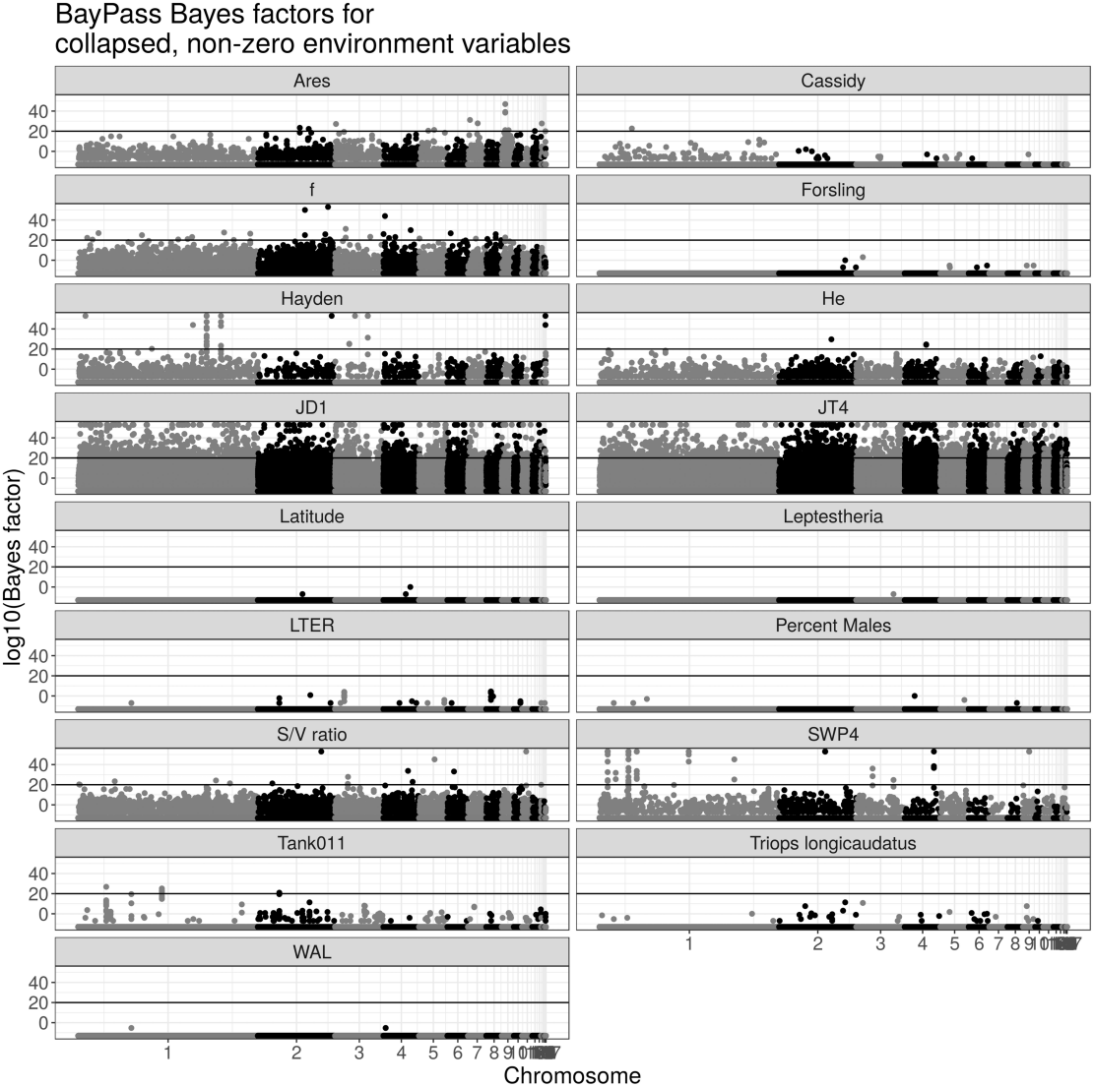
Manhattan plots of single SNP Bayes factors for different environmental variables. All plots show the 11-population Bayes factor associated with the given environmental variable, with environmental variables indicated. Horizontal lines indicate the threshold for the 0.01% most significant loci.

Up to 24 environmental variables (supplementary table 3, Supplementary Material online) were recorded for each population at the time of collection. These include geographic data (latitude, longitude, and elevation), abiotic ecological variables (pond size, pH, etc.), and biotic ecological variables (presence of other species and percentage of males). We used *BayPass*’ Bayes factors to associate these environmental factors with allele frequency differences. To simplify this analysis, we collapsed together environmental variables that were highly correlated across the populations (supplementary fig. 7, Supplementary Material online). We also generated a “dummy” environmental variable for each population (i.e., dummy-LTER has a 1 for LTER, 0 elsewhere; supplementary table 4, Supplementary Material online). Figure 5 depicts Manhattan plots of the Bayes factors for all collapsed variables. Although we attempted to use the same hidden Markov model method that we used for *X^T^X* to instead identify Bayes factor peaks, we found that the large number of near-zero Bayes factor calls, the large degree of variation in genome-wide average Bayes factor between environmental variables, the highly skewed Bayes factor distribution (supplementary table 5, Supplementary Material online), and the observation that many Bayes factor scores are the exact same very small number prevented accurate calling of regions using a hidden Markov model. We ultimately called regions using a cutoff where any Bayes factor above 10^20^ (a highly conservative cutoff—the alternative hypothesis is 10^20^ times more likely than the null hypothesis) was considered significant.

The above complexities make interpretation of the Bayes factor values difficult. Analyses of different environmental variables varied dramatically in their genome-wide average Bayes factor value, with some environmental variables, such as latitude, showing close to no Bayes factors above the lowest level that can be output by the program, whereas other environmental variables, such as the population dummy variables associated with JT4 and JD1, show high Bayes factors across the entire genome. Because so many different, correlated environmental variables were measured, some variables have missing data (e.g., pH, which was only measured in three populations), and populations differed in terms of relatedness to other populations and sequencing coverage, there is likely no single explanation for why a particular environmental variable has a high or low background. That said, some environmental variables are very suggestive outliers. JT4 and JD1, for instance, have by far the largest amount of background, with much of the genome appearing significant. They also are the two most closely related populations (fig. 1). We propose that the variance in allele frequency in a population is at least somewhat higher than *BayPass*’ expectation, and that when two populations are very similar such that *BayPass*’ omega matrix indicates that they should minimally vary in allele frequency, even small deviations between the two populations will produce Bayes factors indicating differentiation between the populations. Several other environmental variables with highly elevated background, including *f*, *He*, and the surface area:volume ratio, are correlated with either the JT4 or JD1 dummy variables. It is also possible that the model is essentially overparameterized given the modest total number of populations being compared.

The population dummy variables for SWP4 and Hayden stand out as having moderate background, and a few regions that have Bayes factor values well above the background. SWP4 has 11 differentiated regions associated with it, 3 of which correspond to differentiated regions found via the *X^T^X* statistic (regions 2, 4, and 13). Hayden has 10 differentiated regions, 7 of which are associated with *X^T^X*-identified regions (regions 1, 8, 9, 14–16, and 19, supplementary table 6, Supplementary Material online). Close inspection of these sites shows that all of these intersecting regions show significantly above-average coverage of the sequence data corresponding to the population (fig. 4 and supplementary fig. 6, Supplementary Material online) as with the *X^T^X* results.

## Discussion

### Natural Populations and Selection

*F*_ST_ analogs are commonly used for detecting local adaptation in model systems. Studies in humans (Mackinnon et al. 2016), *Drosophila* (Reinhardt et al. 2014), and other model organisms (McGaughran et al. 2016) commonly use either whole-genome sequence data (*Drosophila*, other models) or carefully ascertained SNPs from genotyping chips (human and livestock). Given a high-quality genome and large population sample, *F*_ST_-like outliers can detect local adaptation (Savolainen et al. 2013). An increasing number of studies in nonmodel systems have employed a high-quality reference and genotyping data set (e.g., Lamichhaney et al. 2015; McGaughran et al. 2016; Fustier et al. 2017; Leroy et al. 2020). Lamichhaney et al. (2015) sequenced 120 finches across the Galapagos using whole-genome sequencing and a high-quality (5.2 Mb scaffold N50) assembly for alignment. In spite of their restriction to normalized *F*_ST_, which does not account for relationships between populations, they detected several peaks over genes that influence beak morphology. Even so, many of these peaks were as large as the scaffolds they were on, implying poor localization ability. In another example of a study using whole-genome sequencing, McGaughran et al. (2016) sequenced 264 strains of *Pristionchus pacificus* nematodes and performed an *F*_ST_ analysis, identifying locally adapted regions. They went further and demonstrated the functional importance of an *NHX* ortholog in one region. These two studies required the sequencing of a large number of individuals from a broad geographic area, and the construction of a high qualify reference genome. Both identified regions important in local adaptation using *F*_ST_.

Still, scans for local adaptation in nonmodel systems are often performed with fragmented references and sparse genotyping data sets. A low contiguity genome assembly can prevent researchers from identifying peaks of significance. If a peak is larger than the contig that contains it, multiple sections of the peak may be identified as separate peaks. With a high-quality assembly (e.g., Lamichhaney et al. 2015), this problem can still occur in smaller contigs. With no reference at all, as in Lal et al. (2016), SNPs must be analyzed independently, and true local adaptation cannot be distinguished from hitchhiking. In our work, 3 of the 19 *X^T^X* differentiated regions (17–19) are likely artifacts as they are associated with contigs of dubious quality. It is likely that a fragmented assembly would magnify this source of false positives.

**Fig. 6.**
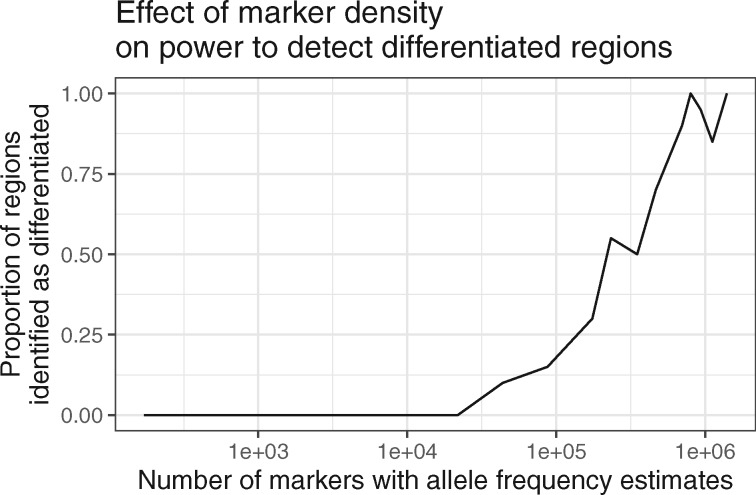
Downsampling of SNPs demonstrates that reduced marker density dramatically reduces power to detect differentiated regions.

If a small number of markers are assayed using a diversity reduction method for genotyping it is possible to completely miss a differentiated peak. Many of our peaks were tagged by between one and a few dozen SNPs despite deep sequencing. This is likely to also be the case in other systems in which LD only extends over short physical distances. Many nonmodel systems have had populations sequenced using candidate gene sequencing (Keller et al. 2012), RADseq (Lal et al. 2016), targeted genomic sequencing (Holliday et al. 2016; Roulin et al. 2016; Yeaman et al. 2016), and other methods (Riginos et al. 2016; Wenzel et al. 2016). Although these techniques are reliable and affordable, they achieve cost efficiency by randomly sampling a subset of SNPs in the genome. We queried a total of 1.4 million SNPs for our *X^T^X* scan, giving us an average resolution of one marker every 85 basepairs. To test if diversity reduction sequencing would result in lower power to identify regions showing population differentiation, we downsampled our empirical data, then applied the same differentiation detecting hidden Markov model to the *X^T^X* values from the downsampled data. In total, 100% of peaks were detected with 1 million markers, 55% with 250 thousand markers (1/4 diversity reduction), and 12% with 100 thousand markers (1/10 diversity reduction; fig. 6). This lends some support to the idea that genotyping without deep sequencing is likely to miss sites that show a signal of population differentiation. Still, it is difficult to generalize because power will depend on levels of LD in the study system. Naturally, coverage should also affect the rate of differentiated region detection (cf., Ferretti et al 2013).

In most studies, *F*_ST_-like statistics are computed using population-wide allele frequency data. Most use *F*_ST_ or a derivative (Lamichhaney et al. 2015; McGaughran et al. 2016), but others use more complex statistics such as those of *BayEnv* (Keller et al. 2012), *Bayescan* (Foll and Gaggiotti 2008; Keller et al. 2012; Lal et al. 2016), *LOSITAN* (Antao et al. 2008; Lal et al. 2016), or others. We used *F*_ST_ and *BayPass* here and found that they identified several of the same genomic regions as being differentiated between the populations, but where they disagree, we believe *BayPass* should be more trustworthy due to its awareness of global population relatedness and incorporation of sequencing depth into significance calculations.

### Local Adaptation Can Be Detected, but Not Characterized, without Ecological Data; Some Questions Remain

Historical studies have successfully correlated allele frequency differences with environmental variables when a general test for elevated *F*_ST_ among all population samples failed to be significant (cf., Berry and Kreitman 1993). In contrast, we identified a set of differentiated regions using only an environment-agnostic measure of population differentiation (*X^T^X*). This demonstrates that modern statistical techniques, combined with whole-genome SNP discovery and analysis, can detect differentially selected sites without knowledge of an organism’s ecology. We also performed environment-aware analyses of allele frequency differentiation (*BayPass*’ Bayes factors) but were unable to distinguish particular environmental variables as being the drivers of population differentiation here. Our results suggest that if the number of populations examined is not considerably larger than the number of ecological variables measured on each population, it is difficult to identify potential ecological causes of adaptation.

One caveat here is the presence of high sequencing coverage in our detected peaks. Because the *X^T^X* statistic is elevated when coverage is high, regions with especially high coverage may have increased power to detect population differentiation with allele count based methods such as *BayPass*. All else being equal, a region with high coverage is more likely to appear as a highly differentiated region using the *X^T^X* statistic, so it is unsurprising that our differentiated regions have higher-than-average coverage. High coverage may be due to PCR duplication, though our use of *picard-tools*’ deduplication pipeline makes this unlikely. More intriguing is the possibility that the high coverage may be due to repetitive sequences, and indeed, several of the called regions are at locations identified as repeats by *RepeatMasker*. There are numerous examples of copy number polymorphisms, which appear in Illumina data as sites with variable coverage, having real phenotypic effects (Chakraborty et al. 2019), so we do not discount the loci identified here based on this signal of high coverage.

### Suggested Future Investigations

We identified a small number of candidate genes associated with population differentiation. A candidate gene approach to understanding the effects of *GAT1*, *polycystin-1*, *Dh44*, and *nephrin* on salt tolerance in *E. texana* or a model organism could be pursued. Flies may or may not be a suitable model for salinity tolerance, but *Daphnia* water fleas are a deeply studied model organism that use osmoregulatory neck organs similar to those in *E. texana* to manage the salinity of their bodies (Potts and Durning 1980). A Crispr knockout of the ortholog of one of these candidate genes in *Daphnia* (Nakanishi et al. 2014; Kumagai et al. 2017; Hiruta et al. 2018) is an attractive approach to understanding their effect in vernal pool shrimp. Future reductions in the cost of sequencing will allow studies like the one here to be carried out with more populations, allowing the effects of specific populations versus environmental characteristics to be statistically disentangled. A promising approach may be to deeply sample populations that span a large range of salinities to further pin down the effect of candidate genes on salinity tolerance. Additionally, experimental evolution toward salinity tolerance followed by population sequencing could shed further light on this phenotype. The methodology of this article provides an outline for characterizing the genetics of local adaptation in never-before-sequenced species: generate a high-quality draft assembly, Poolseq several populations, and identify population differentiation with population structure corrected statistics.

## Data Availability

Additional files are available at the following URL: wfitch.bio.uci.edu/~tdlong/PapersRawData/BaldwinShrimpPopulation.tar.gz. Additionally, all scripts used for analysis will be made available at the following GitHub page: https://github.com/jgbaldwinbrown/jgbutils. The file “*E. texana* local adaptation supplementary information” contains code fragments, tables, and supplementary figure descriptions and supplementary figures, Supplementary Material online.

## Supplementary Material

evaa120_Supplementary_Data

## Notes

Data deposition: This project has been deposited at the NCBI Sequencing Read Archive and GenBank under the accession “PRJNA352082.”

## References

[evaa120-B1] Akey JM, Zhang G, Zhang K, Jin L, Shriver MD. 2002 Interrogating a high-density SNP map for signatures of natural selection. Genome Res. 12(12):1805–1814. 12466284 10.1101/gr.631202 PMC187574

[evaa120-B2] Antao T, Lopes A, Lopes RJ, Beja-Pereira A, Luikart G. 2008 LOSITAN: a workbench to detect molecular adaptation based on a *F*_st_-outlier method. BMC Bioinf. 9(1):323. 10.1186/1471-2105-9-323 PMC2515854 18662398

[evaa120-B3] Baldwin-Brown JG, Weeks SC, Long AD. 2018 A new standard for crustacean genomes: the highly contiguous, annotated genome assembly of the clam shrimp *Eulimnadia texana* reveals HOX gene order and identifies the sex chromosome. Genome Biol Evol. 10(1):143–156. 29294012 10.1093/gbe/evx280 PMC5765565

[evaa120-B4] Berry A, Kreitman M. 1993 Molecular analysis of an allozyme cline: alcohol dehydrogenase in *Drosophila melanogaster* on the east coast of North America. Genetics 134(3):869–893. 8102342 10.1093/genetics/134.3.869 PMC1205523

[evaa120-B5] Bohonak AJ. 1998 Genetic population structure of the fairy shrimp *Branchinecta coloradensis* (Anostraca) in the Rocky Mountains of Colorado. Can J Zool. 76(11):2049–2057.

[evaa120-B6] Brendonck L. 1996 Diapause, quiescence, hatching requirements: what we can learn from large freshwater branchiopods (Crustacea: Branchiopoda: Anostraca, Notostraca, Conchostraca). Hydrobiologia 320(1–3):85–97.

[evaa120-B7] Burke MK, et al 2010 Genome-wide analysis of a long-term evolution experiment with *Drosophila*. Nature 467(7315):587–590. 20844486 10.1038/nature09352

[evaa120-B8] Chakraborty M, Emerson JJ, Macdonald SJ, Long AD. 2019 Structural variants exhibit widespread allelic heterogeneity and shape variation in complex traits. Nat Commun. 10(1):1–11. 31653862 10.1038/s41467-019-12884-1 PMC6814777

[evaa120-B9] Chen N. 2004 Using RepeatMasker to identify repetitive elements in genomic sequences. Curr Protoc Bioinf. 5(1):4.10.1–4.10.14. 10.1002/0471250953.bi0410s25 19274634

[evaa120-B10] Ferretti L, Ramos‐Onsins SE, Pérez‐Enciso M. 2013 Population genomics from pool sequencing. Mol Ecol. 22(22):5561–5576. 24102736 10.1111/mec.12522

[evaa120-B11] Foll M, Gaggiotti O. 2008 A genome-scan method to identify selected loci appropriate for both dominant and codominant markers: a Bayesian perspective. Genetics 180(2):977–993. 18780740 10.1534/genetics.108.092221 PMC2567396

[evaa120-B12] Frichot E, Schoville SD, Bouchard G, François O. 2013 Testing for associations between loci and environmental gradients using latent factor mixed models. Mol Biol Evol. 30(7):1687–1699. 23543094 10.1093/molbev/mst063 PMC3684853

[evaa120-B13] Fustier M-A, et al 2017 Signatures of local adaptation in lowland and highland teosintes from whole-genome sequencing of pooled samples. Mol Ecol. 26(10):2738–2756. 28256021 10.1111/mec.14082

[evaa120-B14] Futschik A, Schlötterer C. 2010 The next generation of molecular markers from massively parallel sequencing of pooled DNA samples. Genetics 186(1):207–218. 20457880 10.1534/genetics.110.114397 PMC2940288

[evaa120-B15] Gautier M. 2015 Genome-wide scan for adaptive divergence and association with population-specific covariates. Genetics 201(4):1555–1579. 26482796 10.1534/genetics.115.181453 PMC4676524

[evaa120-B16] Goodman SN. 1999 Toward evidence-based medical statistics. 2: the Bayes factor. Ann Intern Med. 130(12):1005–1013. 10383350 10.7326/0003-4819-130-12-199906150-00019

[evaa120-B17] Griffiths AJ, Gelbart WM, Miller JH, Lewontin RC. 1999. Modern genetic analysis. New York: W. H. Freeman.

[evaa120-B18] Günther T, Coop G. 2013 Robust identification of local adaptation from allele frequencies. Genetics 195(1):205–220. 23821598 10.1534/genetics.113.152462 PMC3761302

[evaa120-B19] Hill WG, Weir BS. 1988 Variances and covariances of squared linkage disequilibria in finite populations. Theor Popul Biol. 33(1):54–78. 3376052 10.1016/0040-5809(88)90004-4

[evaa120-B20] Hiruta C, Kakui K, Tollefsen KE, Iguchi T. 2018 Targeted gene disruption by use of CRISPR/Cas9 ribonucleoprotein complexes in the water flea *Daphnia pulex*. Genes Cells 23(6):494–502. 29718583 10.1111/gtc.12589

[evaa120-B21] Hivert V, Leblois R, Petit EJ, Gautier M, Vitalis R. 2018 Measuring genetic differentiation from Pool-seq data. Genetics 210(1):315–330. 30061425 10.1534/genetics.118.300900 PMC6116966

[evaa120-B22] Hofer T, Foll M, Excoffier L. 2012 Evolutionary forces shaping genomic islands of population differentiation in humans. BMC Genomics. 13(1):107. 22439654 10.1186/1471-2164-13-107 PMC3317871

[evaa120-B23] Holliday JA, Zhou L, Bawa R, Zhang M, Oubida RW. 2016 Evidence for extensive parallelism but divergent genomic architecture of adaptation along altitudinal and latitudinal gradients in *Populus trichocarpa*. New Phytol. 209(3):1240–1251. 26372471 10.1111/nph.13643

[evaa120-B24] Keightley PD, Ness RW, Halligan DL, Haddrill PR. 2014 Estimation of the spontaneous mutation rate per nucleotide site in a *Drosophila melanogaster* full-sib family. Genetics 196(1):313–320. 24214343 10.1534/genetics.113.158758 PMC3872194

[evaa120-B25] Keller SR, Levsen N, Olson MS, Tiffin P. 2012 Local adaptation in the flowering-time gene network of balsam poplar, *Populus balsamifera* L. Mol Biol Evol. 29(10):3143–3152. 22513286 10.1093/molbev/mss121

[evaa120-B26] Kumagai H, Nakanishi T, Matsuura T, Kato Y, Watanabe H. 2017 CRISPR/Cas-mediated knock-in via non-homologous end-joining in the crustacean *Daphnia magna*. PLoS One 12(10):e0186112. 29045453 10.1371/journal.pone.0186112 PMC5646780

[evaa120-B27] Lal MM, Southgate PC, Jerry DR, Zenger KR. 2016 Fishing for divergence in a sea of connectivity: the utility of ddRADseq genotyping in a marine invertebrate, the black-lip pearl oyster *Pinctada margaritifera*. Mar Genomics. 25:57–68. 26545807 10.1016/j.margen.2015.10.010

[evaa120-B82505556] Lamichhaney S, et al 2015 Evolution of Darwin’s finches and their beaks revealed by genome sequencing. Nature 518(7539):371–375. 25686609 10.1038/nature14181

[evaa120-B28] Leroy T, et al 2020 Massive postglacial gene flow between European white oaks uncovered genes underlying species barriers. New Phytol. 226(4):1183–1197. 31264219 10.1111/nph.16039 PMC7166129

[evaa120-B29] Li H, Durbin R. 2009 Fast and accurate short read alignment with Burrows–Wheeler transform. Bioinformatics 25(14):1754–1760. 19451168 10.1093/bioinformatics/btp324 PMC2705234

[evaa120-B30] Li H, et al 2009 The Sequence Alignment/Map format and SAMtools. Bioinformatics 25(16):2078–2079. 19505943 10.1093/bioinformatics/btp352 PMC2723002

[evaa120-B31] Long AD, Beldade P, Macdonald SJ. 2007 Estimation of population heterozygosity and library construction-induced mutation rate from expressed sequence tag collections. Genetics 176(1):711–714. 17179075 10.1534/genetics.106.063610 PMC1893025

[evaa120-B32] Lotterhos KE, Whitlock MC. 2014 Evaluation of demographic history and neutral parameterization on the performance of *F*_ST_ outlier tests. Mol Ecol. 23(9):2178–2192. 24655127 10.1111/mec.12725 PMC4228763

[evaa120-B33] Mackinnon MJ, et al 2016 Environmental correlation analysis for genes associated with protection against malaria. Mol Biol Evol. 33(5):1188–1204. 26744416 10.1093/molbev/msw004 PMC4839215

[evaa120-B34] Marroni F, et al 2011 Nucleotide diversity and linkage disequilibrium in *Populus nigra* cinnamyl alcohol dehydrogenase (CAD4) gene. Tree Genet Genomes 7(5):1011–1023.

[evaa120-B35] McGaughran A, et al 2016 Genomic profiles of diversification and genotype–phenotype association in island nematode lineages. Mol Biol Evol. 33(9):2257–2272. 27189551 10.1093/molbev/msw093

[evaa120-B36] McKenna A, et al 2010 The genome analysis toolkit: a MapReduce framework for analyzing next-generation DNA sequencing data. Genome Res. 20(9):1297–1303. 20644199 10.1101/gr.107524.110 PMC2928508

[evaa120-B37] Nakanishi T, Kato Y, Matsuura T, Watanabe H. 2014 CRISPR/Cas-mediated targeted mutagenesis in *Daphnia magna*. PLoS One 9(5):e98363. 24878568 10.1371/journal.pone.0098363 PMC4039500

[evaa120-B38] NCBI Resource Coordinators. 2018 Database resources of the National Center for Biotechnology Information. Nucleic Acids Res. 46:D8–D13. 29140470 10.1093/nar/gkx1095 PMC5753372

[evaa120-B39] Nielsen R, et al 2005 Genomic scans for selective sweeps using SNP data. Genome Rese. 15(11):1566–1575. 10.1101/gr.4252305 PMC1310644 16251466

[evaa120-B40] Pickrell JK, Pritchard JK. 2012 Inference of population splits and mixtures from genome-wide allele frequency data. PLoS Genet. 8(11):e1002967. 23166502 10.1371/journal.pgen.1002967 PMC3499260

[evaa120-B41] Potts WTW, Durning CT. 1980 Physiological evolution in the branchiopods. Comp Biochem Physiol Part B Comp Biochem. 67(3):475–484.

[evaa120-B42] Reinhardt JA, Kolaczkowski B, Jones CD, Begun DJ, Kern AD. 2014 Parallel geographic variation in *Drosophila melanogaster*. Genetics 197(1):361–373. 24610860 10.1534/genetics.114.161463 PMC4012493

[evaa120-B43] Riginos C, Crandall ED, Liggins L, Bongaerts P, Treml EA. 2016 Navigating the currents of seascape genomics: how spatial analyses can augment population genomic studies. Curr Zool. 62(6):581–601. 29491947 10.1093/cz/zow067 PMC5804261

[evaa120-B44] Roulin AC, Bourgeois Y, Stiefel U, Walser J-C, Ebert D. 2016 A photoreceptor contributes to the natural variation of diapause induction in *Daphnia magna*. Mol Biol Evol. 33(12):3194–3204. 27660296 10.1093/molbev/msw200

[evaa120-B45] Sassaman C, Weeks SC. 1993 The genetic mechanism of sex determination in the conchostracan shrimp *Eulimnadia texana*. Am Nat. 141(2):314–328. 19426084 10.1086/285475

[evaa120-B46] Savolainen O, Lascoux M, Merilä J. 2013 Ecological genomics of local adaptation. Nat Rev Genet. 14(11):807–820. 24136507 10.1038/nrg3522

[evaa120-B47] Stanke M, Waack S. 2003 Gene prediction with a hidden Markov model and a new intron submodel. Bioinformatics 19(Suppl 2):ii215–ii225. 14534192 10.1093/bioinformatics/btg1080

[evaa120-B48] Stumpf MPH, McVean G. 2003 Estimating recombination rates from population-genetic data. Nat Rev Genet. 4(12):959–968. 14631356 10.1038/nrg1227

[evaa120-B49] Tajima F. 1989 Statistical method for testing the neutral mutation hypothesis by DNA polymorphism. Genetics 123(3):585–595. 2513255 10.1093/genetics/123.3.585 PMC1203831

[evaa120-B50] Voight BF, Kudaravalli S, Wen X, Pritchard JK. 2006 A map of recent positive selection in the human genome. PLoS Biol. 4(3):e72. 16494531 10.1371/journal.pbio.0040072 PMC1382018

[evaa120-B51] Watterson GA. 1975 On the number of segregating sites in genetical models without recombination. Theor Popul Biol. 7(2):256–276. 1145509 10.1016/0040-5809(75)90020-9

[evaa120-B52] Weeks SC, Benvenuto C, Sanderson TF, Duff RJ. 2010 Sex chromosome evolution in the clam shrimp, *Eulimnadia texana*. J Evol Biol. 23(5):1100–1106. 20298443 10.1111/j.1420-9101.2010.01963.x

[evaa120-B53] Weeks SC, Marcus V, Crosser BR. 1999 Inbreeding depression in a self-compatible, androdioecious crustacean, *Eulimnadia texana*. Evolution 53(2):472–483. 28565429 10.1111/j.1558-5646.1999.tb03782.x

[evaa120-B54] Weeks SC, Zucker N. 1999 Rates of inbreeding in the androdioecious clam shrimp *Eulimnadia texana*. Can J Zool. 77(9):1402–1408.

[evaa120-B55] Weir BS, Cockerham CC. 1984 Estimating *F*-statistics for the analysis of population structure. Evolution 38(6):1358–1370. 28563791 10.1111/j.1558-5646.1984.tb05657.x

[evaa120-B56] Wenzel MA, Douglas A, James MC, Redpath SM, Piertney SB. 2016 The role of parasite-driven selection in shaping landscape genomic structure in red grouse (*Lagopus lagopus scotica*). Mol Ecol. 25(1):324–341. 26578090 10.1111/mec.13473

[evaa120-B57] Yang Z, Bielawski JP. 2000 Statistical methods for detecting molecular adaptation. Trends Ecol Evol. 15(12):496–503. 11114436 10.1016/S0169-5347(00)01994-7 PMC7134603

[evaa120-B58] Yeaman S, et al 2016 Convergent local adaptation to climate in distantly related conifers. Science 353(6306):1431–1433. 27708038 10.1126/science.aaf7812

