# Supplementary material for: Genomic Signatures of Local Adaptation in Clam Shrimp (Eulimnadia texana) from Natural Vernal Pools
Source: Genome Biol Evol. 2020 Jun 15;12(7):1194–206. doi: 10.1093/gbe/evaa120 (PMC7486962; doi:10.1093/gbe/evaa120)
Supplement: evaa120_Supplementary_Data [file evaa120_supplementary_data.zip › Supplementary_figures.pdf]

## 0.1 Supplementary figures

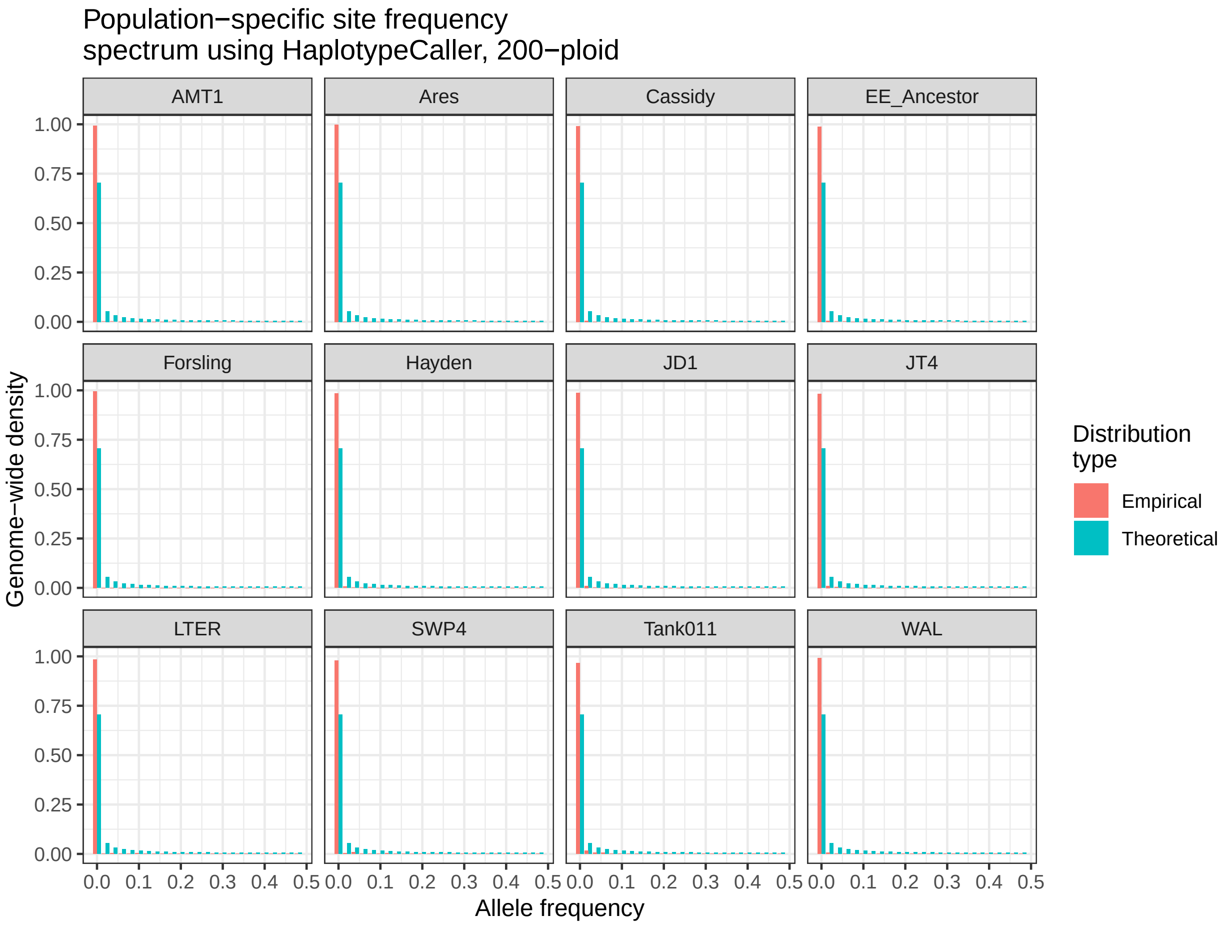


Sup. Fig. 1

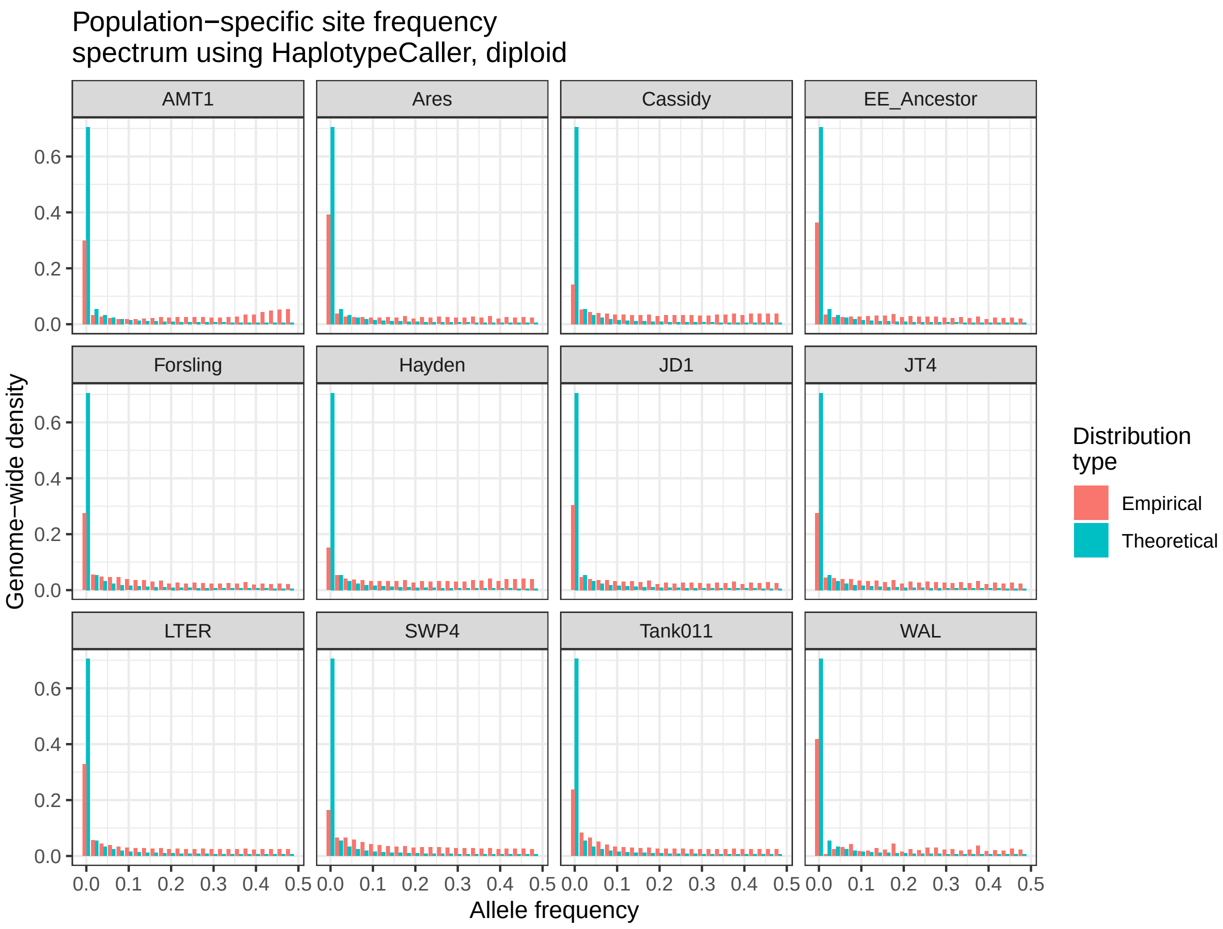
Population−specific site frequency
spectrum using HaplotypeCaller, diploid
AMT1
Ares
Cassidy
EE_Ancestor
Forsling
Hayden
JD1
JT4
LTER
SWP4
Tank011
WAL
0.0
0.2
0.4
0.6
0.0
0.1
0.2
0.3
0.4
0.5
Genome−wide density
Allele frequency
Distribution type
Empirical
Theoretical

Sup. Fig. 2

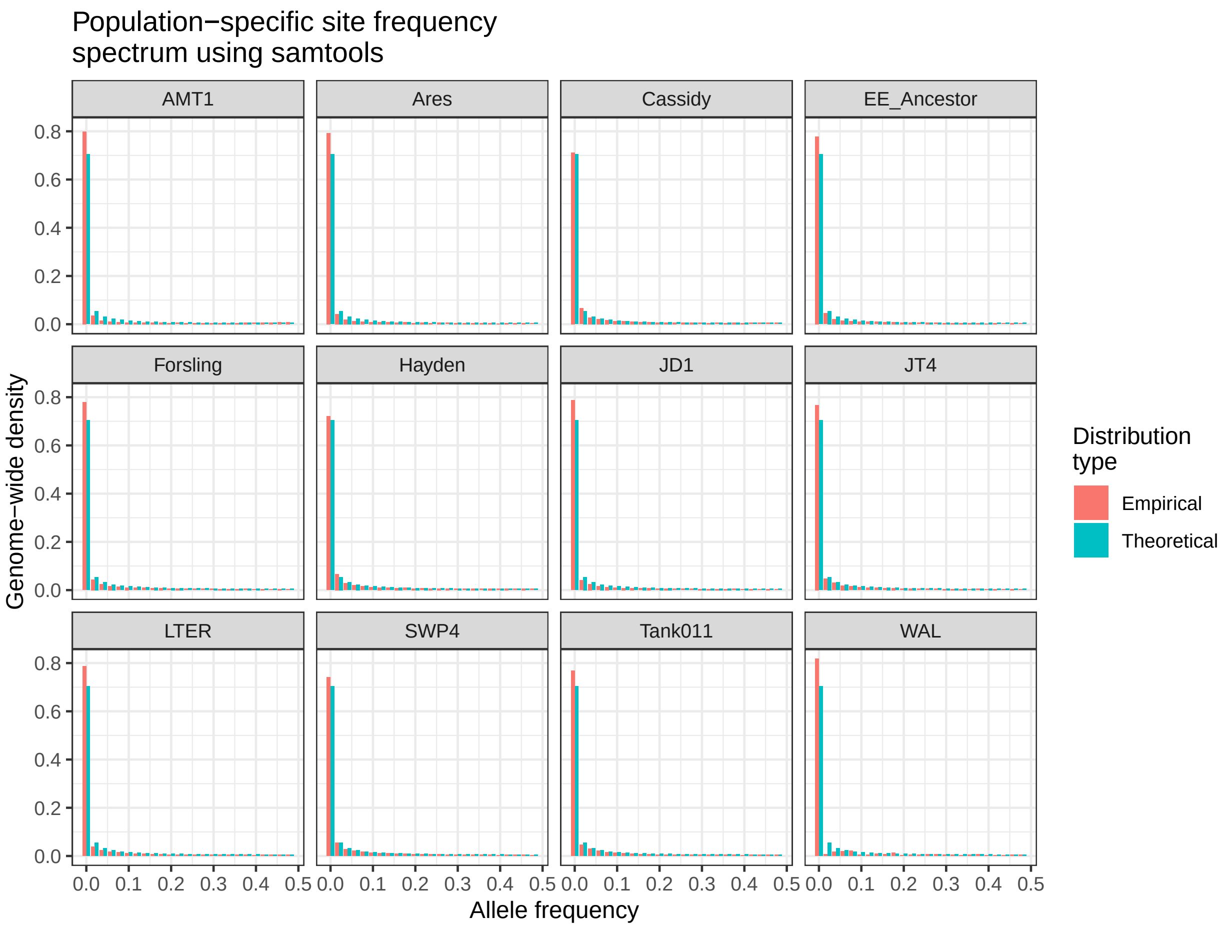
Population−specific site frequency
spectrum using samtools
AMT1
Ares
Cassidy
EE_Ancestor
Forsling
Hayden
JD1
JT4
LTER
SWP4
Tank011
WAL
0.0
0.2
0.4
0.6
0.8
0.0 0.1 0.2 0.3 0.4 0.5
Genome−wide density
Allele frequency
Distribution type
Empirical
Theoretical

Sup. Fig. 3

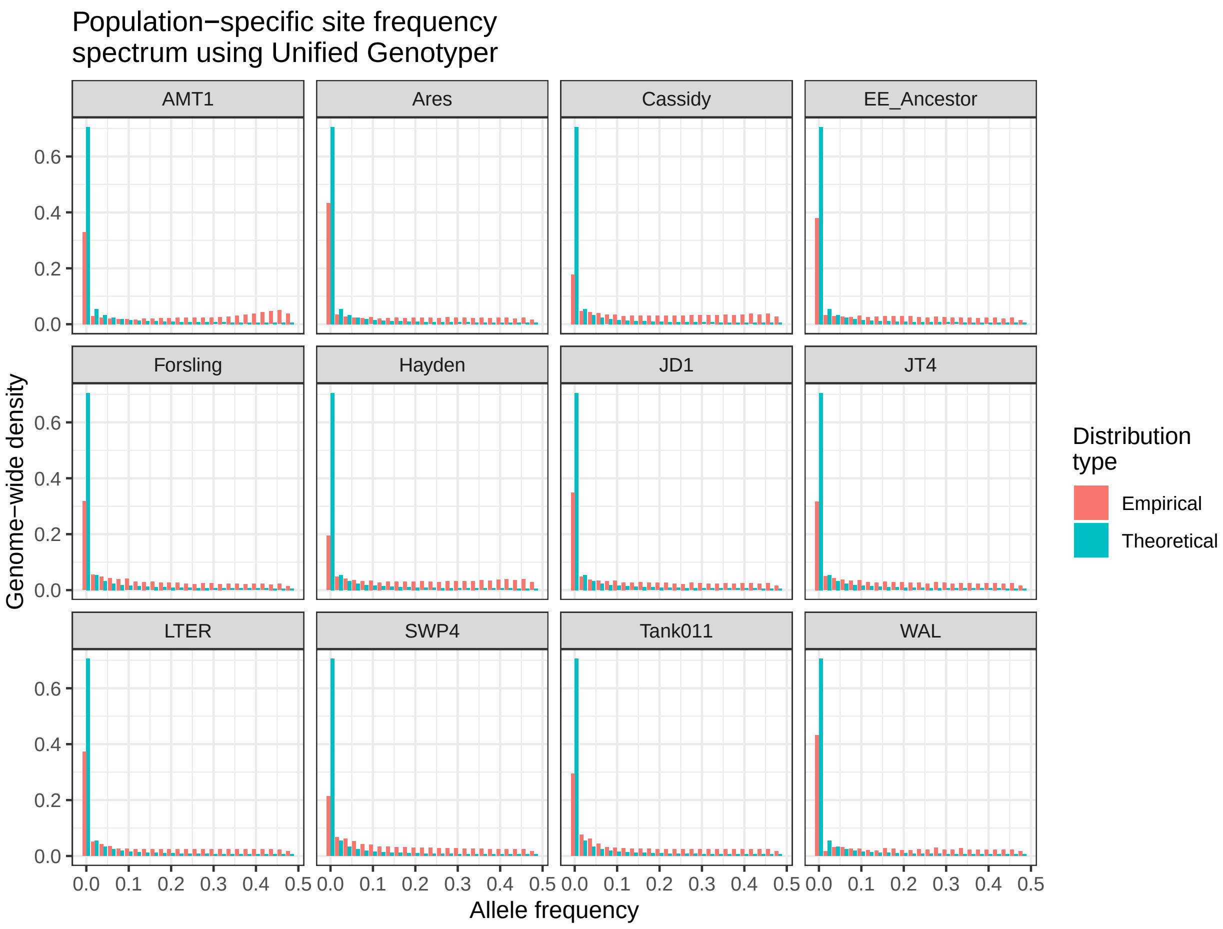
Population−specific site frequency
spectrum using Unified Genotyper
AMT1
Ares
Cassidy
EE_Ancestor
Forsling
Hayden
JD1
JT4
LTER
SWP4
Tank011
WAL
Genome−wide density
Allele frequency
Distribution
type
Empirical
Theoretical

Sup. Fig. 4

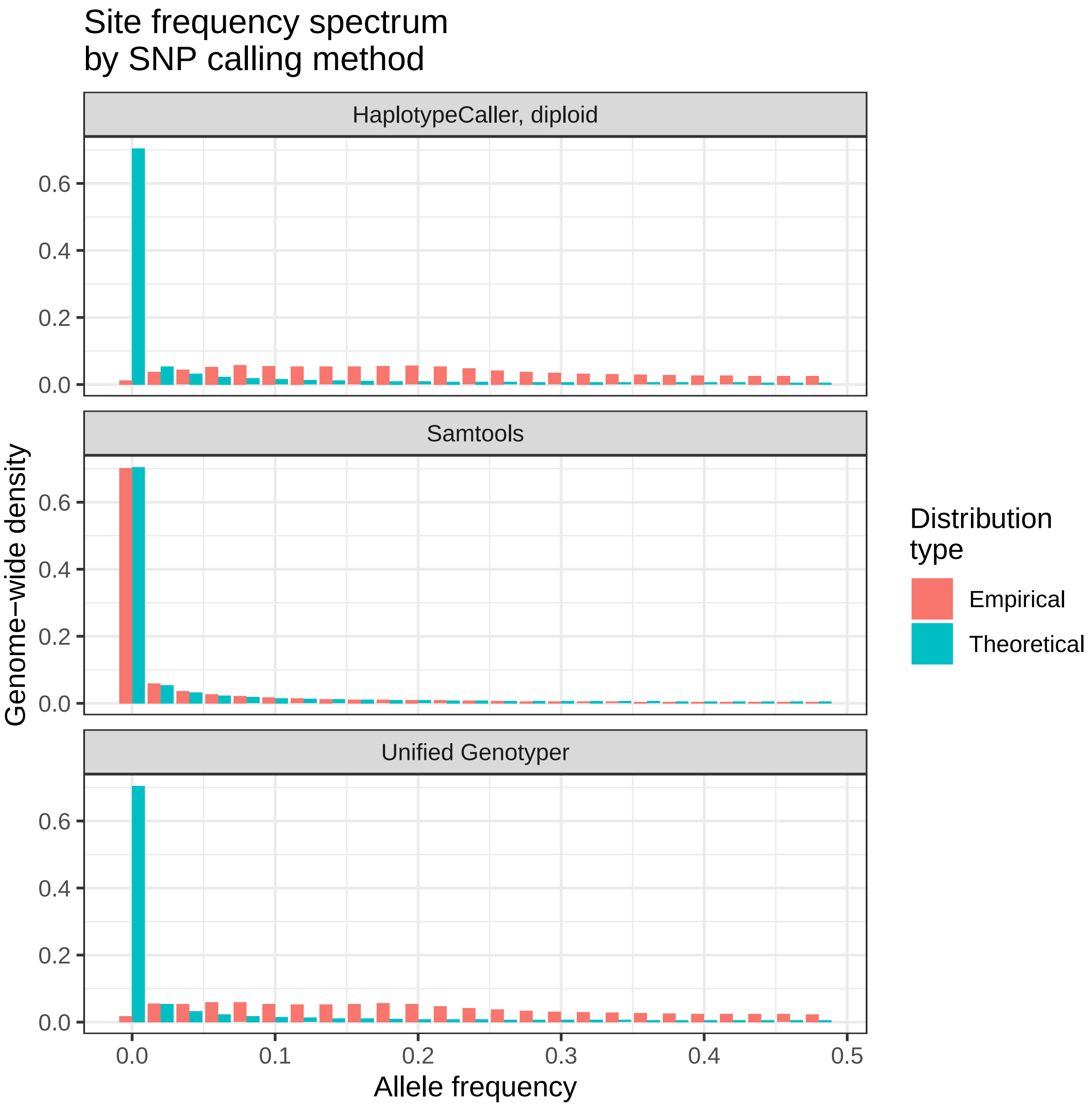
Site frequency spectrum
by SNP calling method
HaplotypeCaller, diploid
Samtools
Unified Genotyper
Genome−wide density
0.0
0.2
0.4
0.6
0.0
0.1
0.2
0.3
0.4
0.5
Allele frequency
Distribution
type
Empirical
Theoretical

Sup. Fig. 5

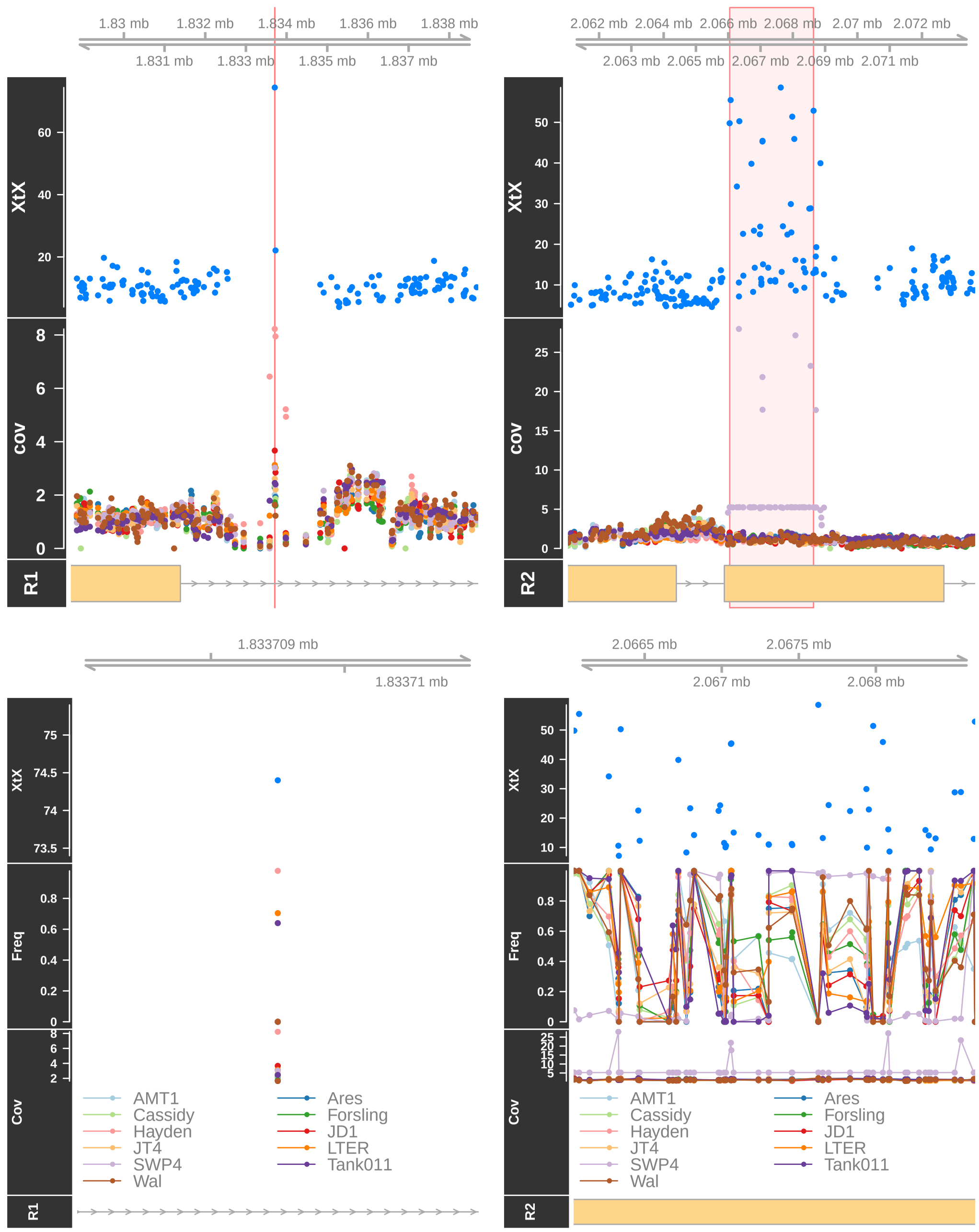


Sup. Fig. 6. Continued

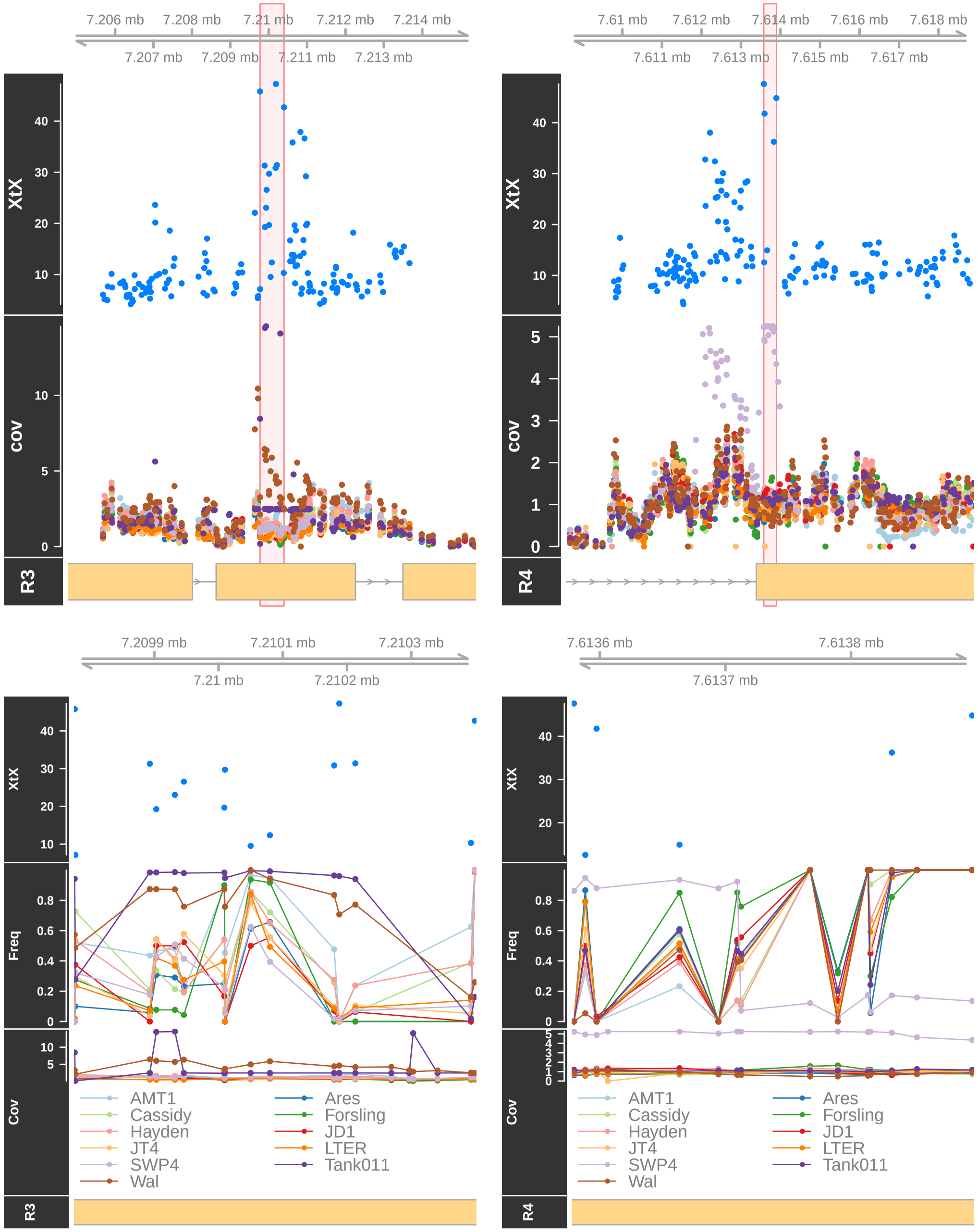


Sup. Fig. 6. Continued

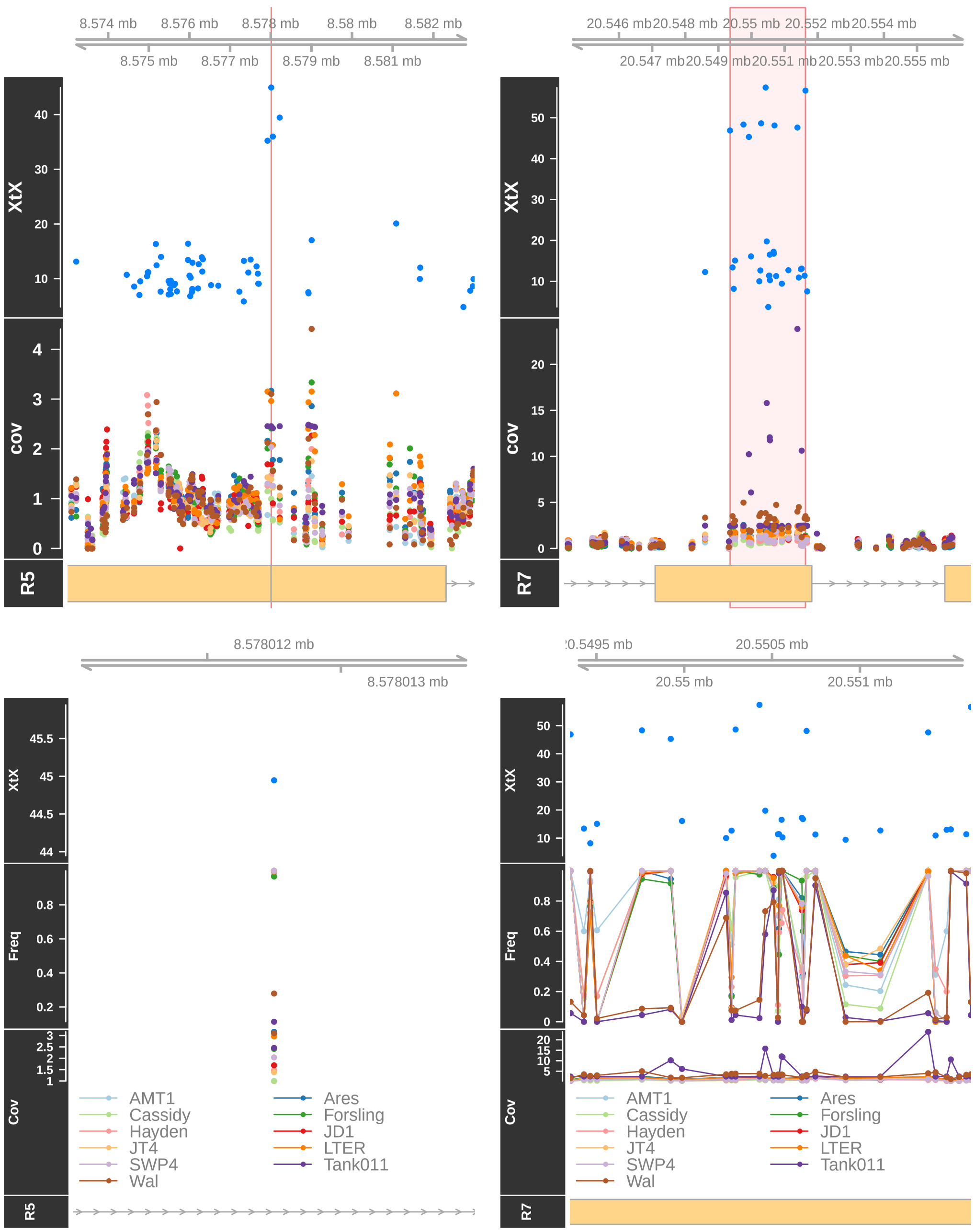


Sup. Fig. 6. Continued

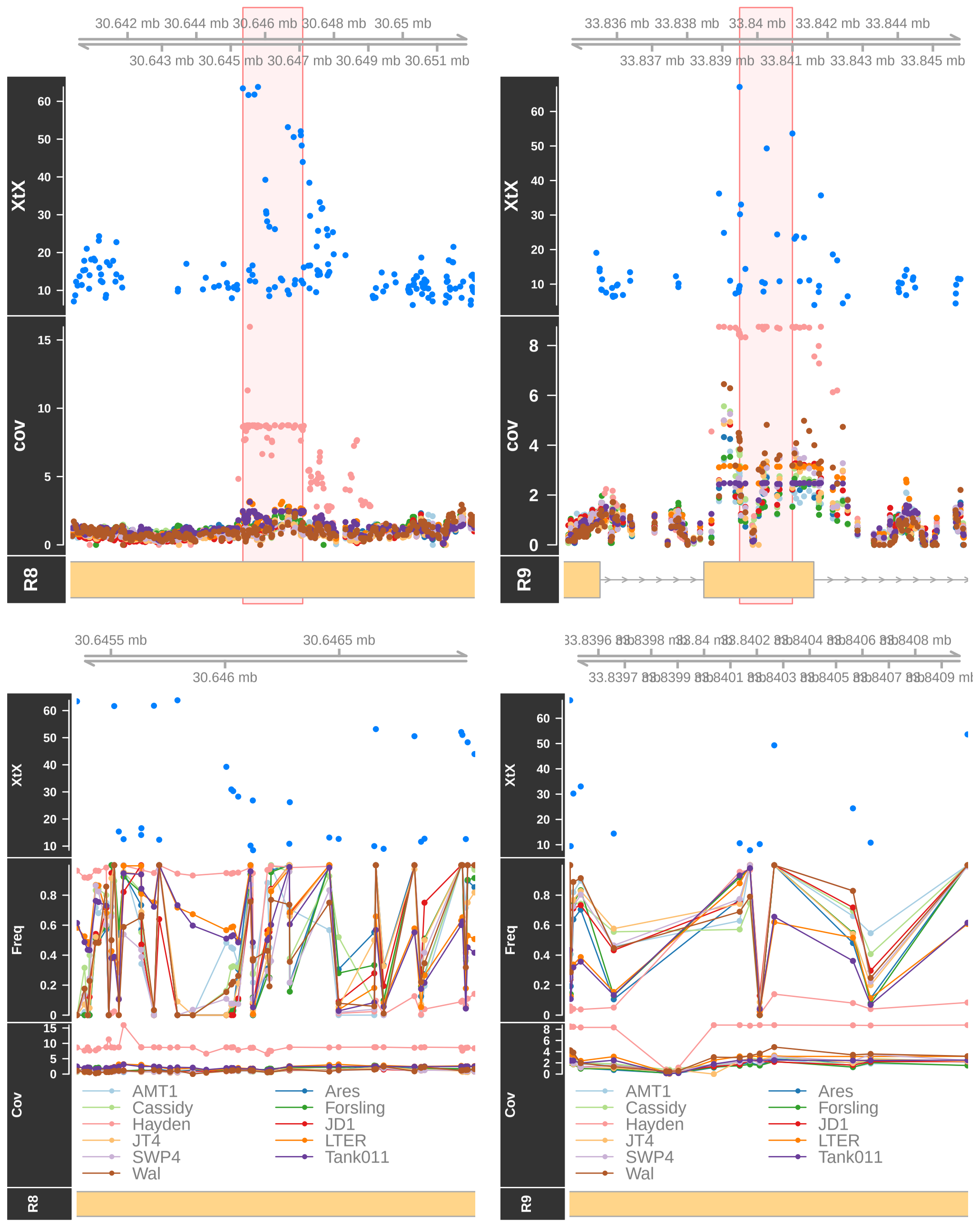


Sup. Fig. 6. Continued

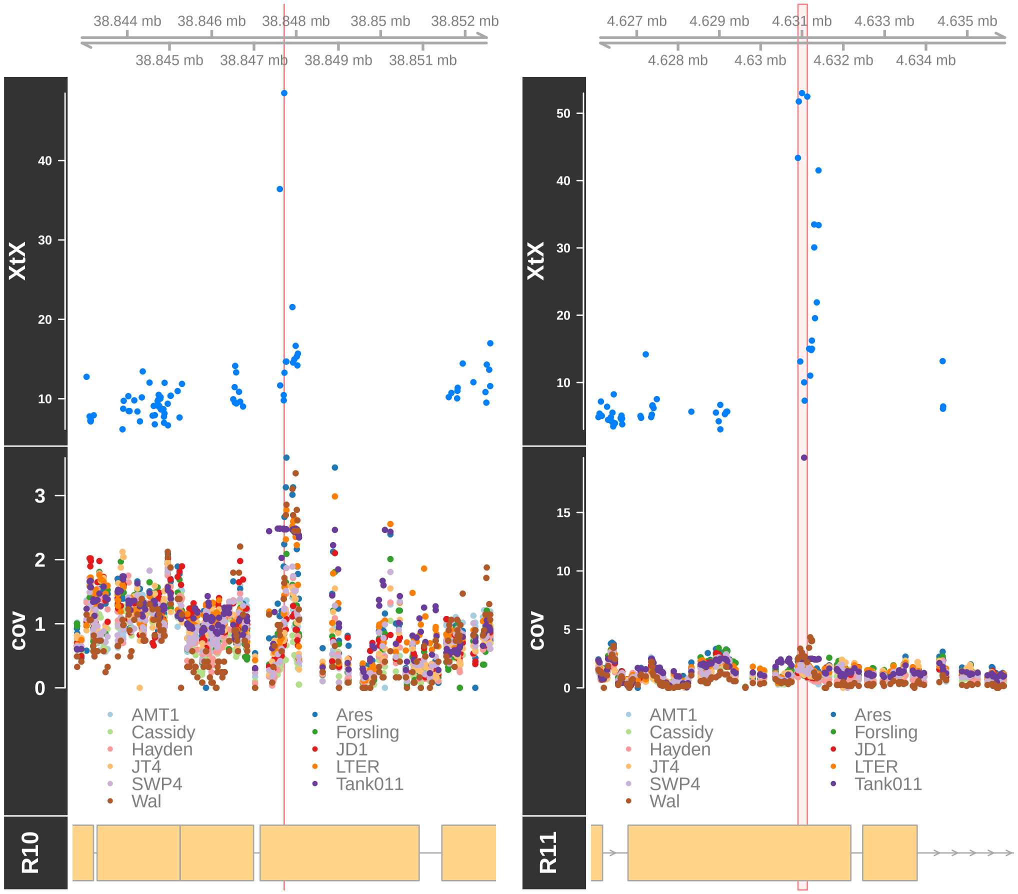


Sup. Fig. 6. Continued

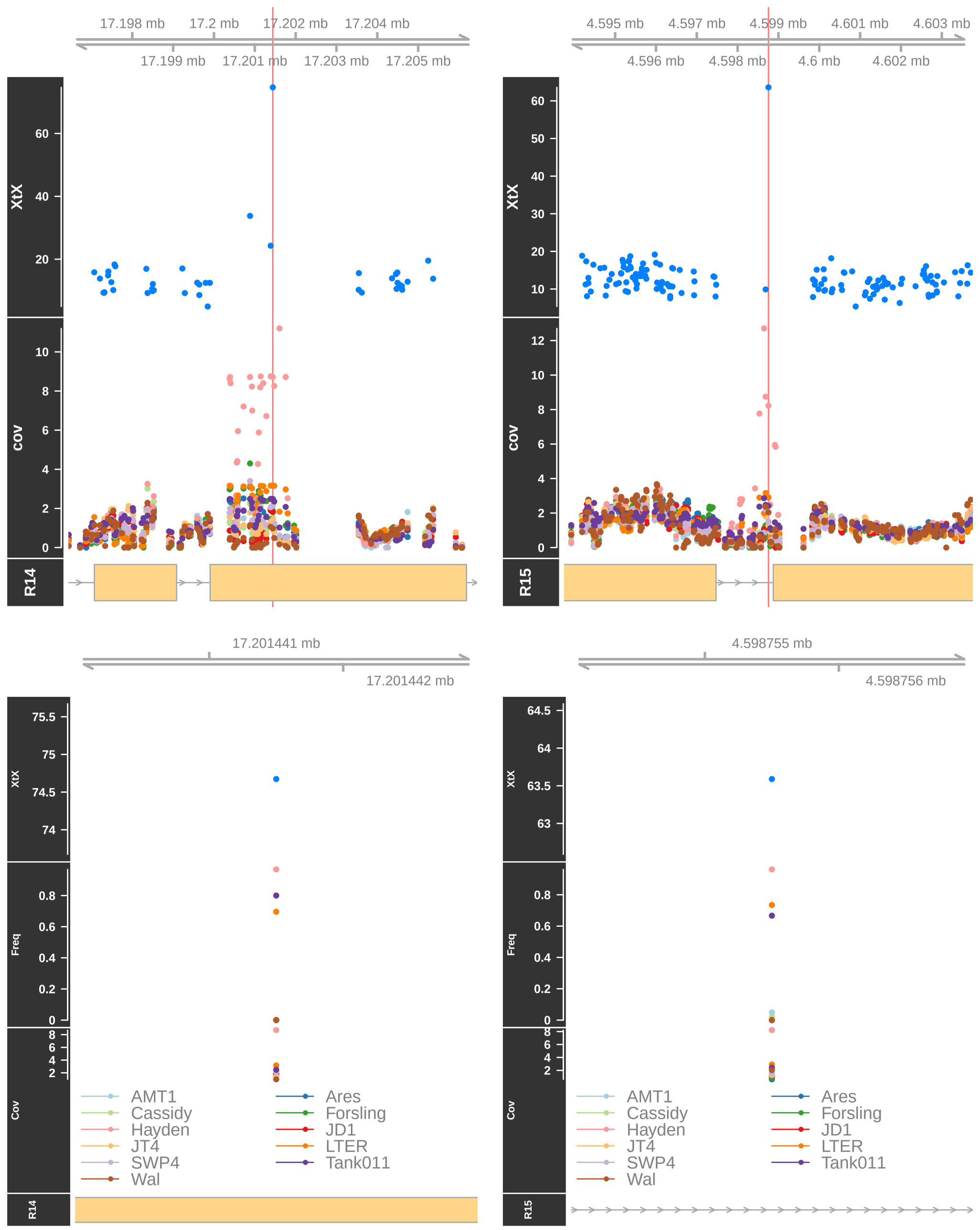


Sup. Fig. 6. Continued

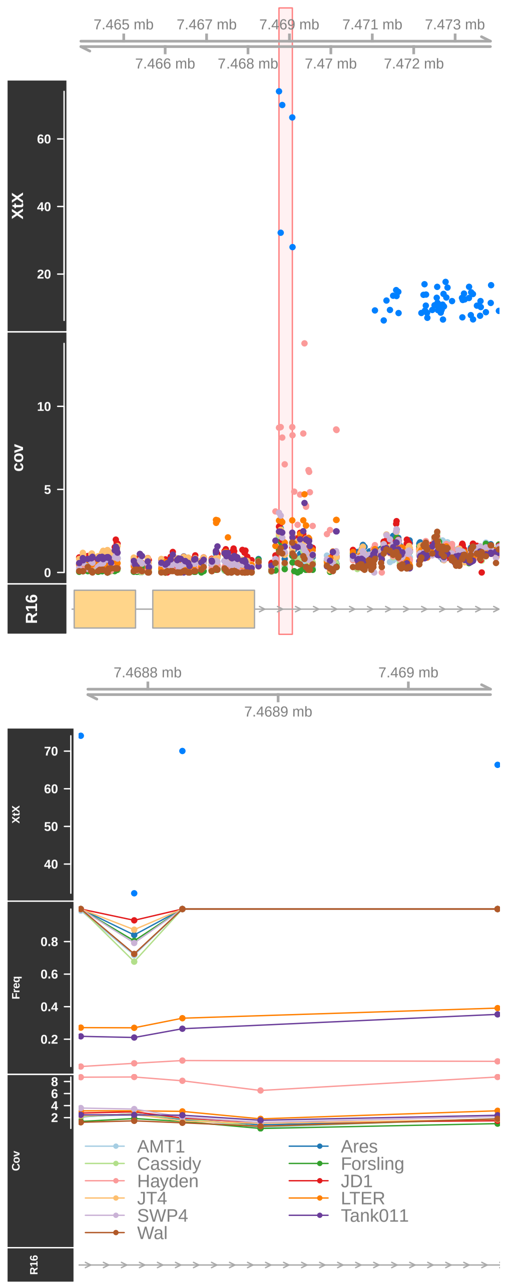


Sup. Fig. 6. Continued

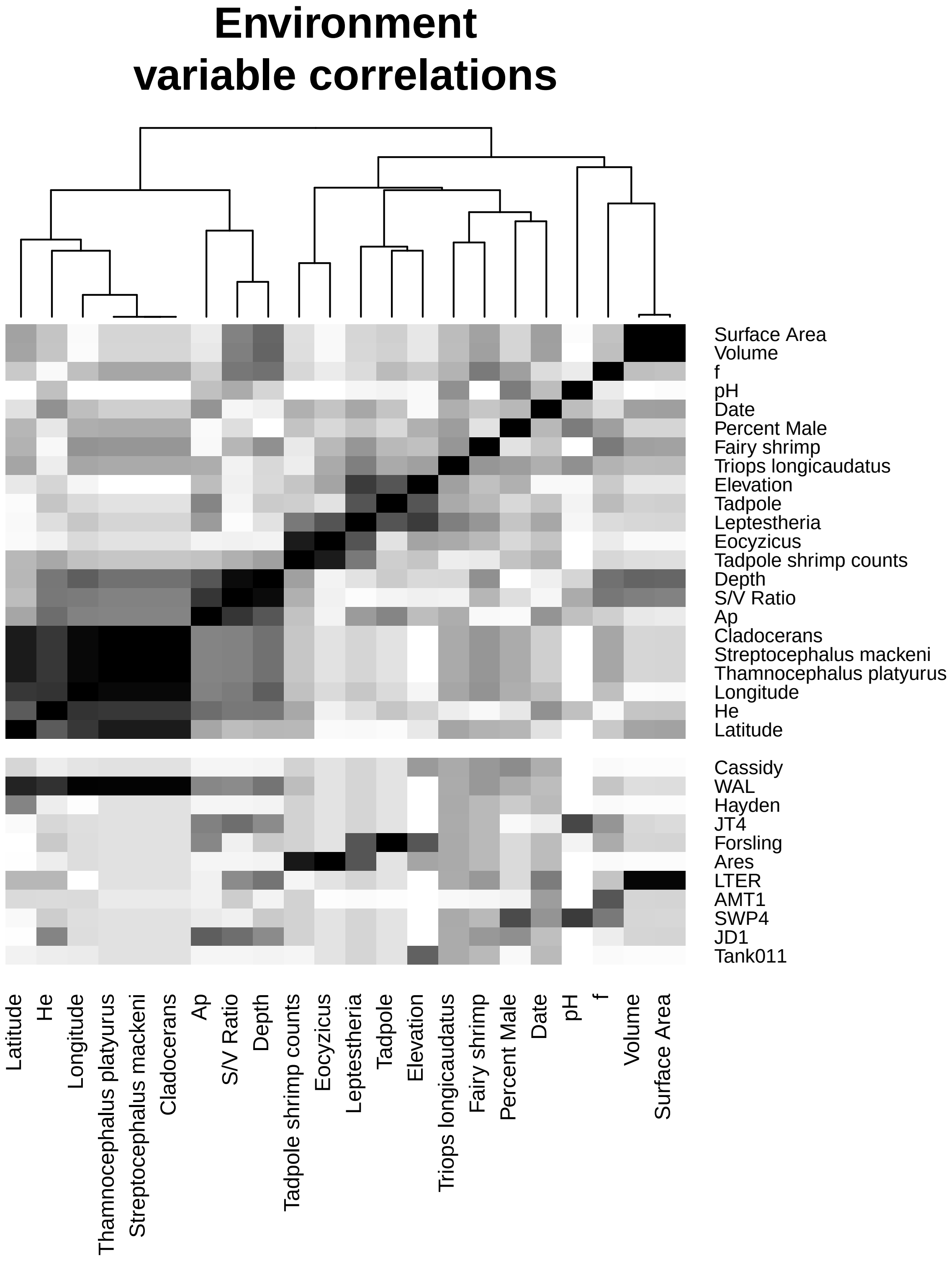
Environment
variable correlations
Surface Area
Volume
f
pH
Date
Percent Male
Fairy shrimp
Triops longicaudatus
Elevation
Tadpole
Leptestheria
Eocyzicus
Tadpole shrimp counts
Depth
S/V Ratio
Ap
Cladocerans
Streptocephalus mackeni
Thamnocephalus platyurus
Longitude
He
Latitude
Cassidy
WAL
Hayden
JT4
Forsling
Ares
LTER
AMT1
SWP4
JD1
Tank011
Latitude
He
Longitude
Thamnocephalus platyurus
Streptocephalus mackeni
Cladocerans
Ap
S/V Ratio
Depth
Tadpole shrimp counts
Eocyzicus
Leptestheria
Tadpole
Elevation
Triops longicaudatus
Fairy shrimp
Percent Male
Date
pH
f
Volume
Surface Area

Sup. Fig. 7
