# Supplementary material for: Genomic Signatures of Local Adaptation in Clam Shrimp (Eulimnadia texana) from Natural Vernal Pools
Source: Genome Biol Evol. 2020 Jun 15;12(7):1194–206. doi: 10.1093/gbe/evaa120 (PMC7486962; doi:10.1093/gbe/evaa120)
Supplement: evaa120_Supplementary_Data [file evaa120_supplementary_data.zip › Supplementary_information_clean.docx]

# Supplement

## Supplementary texts

### SNP calling

As noted in the main text, we explored four different methods to obtain allele frequencies at SNPs. Of these, we settled on GATK’s Diploid HaplotypeCaller (called DHC below) because it has been empirically demonstrated to provide more accurate SNP calls than either *samtools* or UnifiedGenotyper (Pirooznia et al. 2014).

We explored three alternate SNP calling pipelines and excluded them for the reasons stated below:

*GATK*’s *UnifiedGentoyper:* Our UnifiedGenotyper pipeline for cleaning Illumina sequencing data, aligning to the reference, and calling SNPs was as follows: align using *BWA* 0.7.8 (Li and Durbin 2009), deduplicate data using *Picard tools* 1.130 (<https://sourceforge.net/projects/picard/>), and call SNPs using *GATK* 3.8 (McKenna et al. 2010). GATK filters reads based on mapped quality, with minimum quality of 30 for inclusion in the data. SNP read depth was required to be at least 10 per pool. After SNP calling, we censored SNPs by coverage using the following protocol: after merging the WAL and EE populations, remove all SNPs that have a mapped coverage of less than 10 or more than 200 in any population (in the two deeply sequenced samples, the 200 cutoff was applied to the coverages after random down-sampling of reads to match the lower coverage populations), and remove all SNPs that, in any population, have a coverage more than 3 standard deviations from the population’s mean coverage. We performed this censoring separately for each of the three population comparisons (WAL vs. EE, Tank011 vs. LTER, and all-population) discussed below. This left a total of 1.4 million SNPs. Although the SNP calls produced by UnifiedGenotyper were ultimately similar in SFS to those produced by the HaplotypeCaller pipeline (Sup. Fig 5), we chose to use the HaplotypeCaller SNP calls because of empirically demonstrated higher SNP calling accuracy with HaplotypeCaller (Pirooznia et al. 2014).

*GATK*’s *HaplotypeCaller* (200-ploid): The 200-ploid HaplotypeCaller polymorphism calling was performed due to GATK’s preference for discarding rare polymorphisms under the default diploid setting. We ran GATK under the same settings used for the diploid HaploCaller run described in the main text, except set the “--ploidy” switch to 200. Censoring was identical to the diploid HaploCaller procedure. We discarded this method because GATK under these conditions called only 464 sites as polymorphic, and the SFS of these sites was highly irregular (Sup. Fig. 5).

*mpileup* in *samtools*:

We performed the samtools procedure with the following commands:

samtools mpileup \

-t DP \

-f data/ref/shrimp_qmerged.quiver.fasta \

-b data/bamsfile.txt | \

pigz -p 6 \

> inter/full_mpileup_all.mpileup.gz

mkfifo fif

java -jar progs/mpileup2sync.jar \

--input <(gunzip -c inter/full_mpileup_all.mpileup.gz) \

--output fif \

--min-qual 30 \

--threads 6 &

cat fif | gzip -c > inter/full_sync_all.sync.gz

rm fif

### This generated a samtools mpileup file containing all of the data, and then converted it into a *popoolation* syncfile for tabulating allele frequencies. Only loci that had at least 1 polymorphic allele and a coverage of at least 10 read pairs per population were kept after censoring. *samtools*’ (Li et al. 2009) mpileup tool produced a neutral-looking site frequency spectrum, but the large number of rare SNPs could not be realistically handled by *BayPass.* This pipeline identified 20,162,092 polymorphic sites, but censoring by coverage reduced this number to 11,214,885, or 8.5 times more polymorphisms than the diploid HaplotypeCaller pipeline after censoring. 83% of the SNPs used in our final pipeline were shared with the *samtools* pipeline. As the SFS demonstrates (Sup. Fig. 5), most of the polymorphisms identified by *samtools* but missing in the diploid HaplotypeCaller pipeline are rare, cannot be easily accommodated in the *BayPass* software, and unlikely to show allele frequency differences between populations at any rate. Thus, a diploid HaplotypeCaller run produced the most accurate allele counts for any given polymorphism, and preferentially included the polymorphisms most likely to influence the results.

Supplementary Figures 1-5 plot the observed minor allele frequency spectrum and the expected allele frequency spectrum under neutrality. Supplementary figures 1-4 show the allele frequency spectrum for each population under all four of our SNP calling regimes, while supplementary figure 5 shows the allele frequency spectrum of the aggregate of all population for each of the four regimes. The expected neutral allele frequency spectrum contains a large number of rare alleles that are not identified by our SNP calling pipeline (HaplotypeCaller, diploid), likely because our pipeline mis-identifies some true SNPs as sequencing errors.

### Calculation of population genetics statistics

Watterson’s θ (Watterson 1975) is biased when SNP ascertainment is imperfect. This is potentially an acute problem with Poolseq datasets. Rare alleles are underrepresented when SNP are censored that are below an arbitrary frequency cut-off in the pool (e.g. minor alleles at a frequency of <2% in the pool are routinely culled), but rare alleles will be over-counted if no cut-off is employed (and Illumina sequencing errors are erroneously called as SNPs). We estimated theta using *npstat* (Ferretti et al. 2013) and an estimator based only on common SNPs with a correction for the ascertainment process (Long et al. 2007). The ascertainment corrected estimate of theta accounts for the ascertainment bias by fitting the neutral site frequency spectrum expected by chance (Fu 1995) to SNPs with minor allele frequency above 0.1 (and thus unlikely to be sequencing errors) using code available on GitHub (<https://github.com/jgbaldwinbrown/jgbutils>). We calculated pi using closely related code also available on GitHub.

We similarly calculated ρ by first estimating the short-distance linkage disequilibrium using *LDx* (Feder, Petrov, and Bergland 2012) for the same set of non-censored SNPs obtained from the DHC pipeline. We then estimated ρ by modeling decay of linkage disequilibrium (*r*^2^) with distance in basepairs using a non-linear model, as in Marroni et al. 2011.

### Collapsing environmental variables

We correlated each dummy variable with each environmental variable and collapsed environmental variables with an absolute *𝜚* greater than 95% with any dummy variable. This data reduction resulted in the following collapses: *Thamnocephalus platyurus*, *Streptocephalus mackeni*, and Cladoceran presence/absence were collapsed to WAL; *Eocyzicus* presence/absence was collapsed to Ares; Tadpole shrimp presence/absence collapsed to Forsling; volume and surface area collapsed to LTER. Finally, the surface area-to-volume ratio and depth were collapsed. This reduced the number of environmental variables from 24 to 13 (Sup. Table 4).

### Settings

*BWA* 0.7.8 and *samtools* 1.9 command line settings for aligning and filtering reads were as follows:

bwa aln -t ${CORES} \

$REFPATH \

$FDATAPATH \

> ${OUTPATH}.F.sai
bwa aln -t ${CORES} \

$REFPATH \

$RDATAPATH \

> ${OUTPATH}.R.sai
bwa sampe ${REFPATH} \

${OUTPATH}.F.sai \

${OUTPATH}.R.sai \

$FDATAPATH $RDATAPATH | \

samtools view -q 20 -bS - | \

samtools sort - data/bam/$PREFIX

*picard-tools* 1.96 command line settings for deduplication were as follows:

java -jar picard.jar \

MarkDuplicates \

INPUT=${prefix}.bam \

OUTPUT=${prefix}.dedup.bam \

METRICS_FILE=${prefix}.dedup.metrics.txt \

REMOVE_DUPLICATES=true}

*GATK* 3.1.1 command line settings for calling SNPs were as follows:

java -d64 -Xmx128g \

-jar GenomeAnalysisTK.jar \

-T UnifiedGenotyper \

-nt ${CORES} \

-R ${REFPATH} \

-I merged-realigned-deduped.bam \

-gt_mode DISCOVERY \

-stand_call_conf 30 \

-stand_emit_conf 10 \

-o rawSNPS-Q30_v2.vcf

java -d64 -Xmx128g \

-jar GenomeAnalysisTK.jar \

-T VariantAnnotator \

-nt ${CORES} \

-R ${REFPATH} \

-I merged-realigned-deduped.bam \

-G StandardAnnotation \

-V:variant,VCF rawSNPS-Q30_v2.vcf \

-XA SnpEff \

-o rawSNPS-Q30-annotated_v2.vcf

java -d64 -Xmx128g \

-jar GenomeAnalysisTK.jar \

-T UnifiedGenotyper \

-nt ${CORES} \

-R ${REFPATH} \

-I merged-realigned-deduped.bam \

-gt_mode DISCOVERY \

-glm INDEL \

-stand_call_conf 30 \

-stand_emit_conf 10 \

-o inDels-Q30_v2.vcf

java -d64 -Xmx20g \

-jar GenomeAnalysisTK.jar \

-T VariantFiltration \

-R ${REFPATH} \

-V rawSNPS-Q30-annotated_v2.vcf \

--mask inDels-Q30_v2.vcf \

--maskExtension 5 \

--maskName InDel \

--clusterWindowSize 10 \

--filterExpression "MQ0 >= 4 && ((MQ0 / (1.0 * DP)) > 0.1)" \

--filterName "BadValidation" \

--filterExpression "QUAL < 30.0" \

--filterName "LowQual" \

--filterExpression "QD < 5.0" \

--filterName "LowVQCBD" \

--filterExpression "FS > 60" \

--filterName "FisherStrand" \

-o Q30-SNPs_v2.vcf

cat Q30-SNPs_v2.vcf | \

grep 'PASS|textasciicircum#' \

> only-PASS-Q30-SNPs_v2.vcf

java -d64 -Xmx20g \

-jar GenomeAnalysisTK.jar \

-T VariantFiltration \

-R ${REFPATH} \

-V inDels-Q30_v2.vcf \

--clusterWindowSize 10 \

--filterExpression "MQ0 >= 4 && ((MQ0 / (1.0 * DP)) > 0.1)" \

--filterName "BadValidation" \

--filterExpression "QUAL < 30.0" \

--filterName "LowQual" \

--filterExpression "QD < 5.0" \

--filterName "LowVQCBD" \

--filterExpression "FS > 60" \

--filterName "FisherStrand" \

-o Q30-INDEL_v2.vcf

cat Q30-INDEL_v2.vcf | \

grep 'PASS|textasciicircum#' \

> only-PASS-Q30-INDEL_v2.vcf

*GATK* 3.7 command line settings for calling SNPs with HaplotypeCaller were as follows:

java -d64 -Xmx15g \

-jar /data/apps/gatk/3.7/GenomeAnalysisTK.jar \

-T HaplotypeCaller \

-R ${REFPATH} \

-I ../merged-realigned-deduped2.bam \

-gt_mode DISCOVERY \

-stand_call_conf 30 \

-o rawSNPS-Q30_v2.vcf

java -d64 -Xmx15g \

-jar /data/apps/gatk/3.7/GenomeAnalysisTK.jar \

-T VariantAnnotator \

-nt ${CORES} \

-R ${REFPATH} \

-I ../merged-realigned-deduped2.bam \

-G StandardAnnotation \

-V:variant,VCF rawSNPS-Q30_v2.vcf \

-XA SnpEff \

-o rawSNPS-Q30-annotated_v2.vcf

java -d64 -Xmx15g \

-jar /data/apps/gatk/3.7/GenomeAnalysisTK.jar \

-T VariantFiltration \

-R ${REFPATH} \

-V rawSNPS-Q30-annotated_v2.vcf \

--clusterWindowSize 10 \

--filterExpression "QUAL < 30.0" --filterName "LowQual" \

--filterExpression "QD < 5.0" --filterName "LowVQCBD" \

--filterExpression "FS > 60.0" --filterName "FisherStrand" \

-o Q30-SNPs_v2.vcf

## Supplementary tables

| Population | Oligo used | Adapter sequence | Index sequence |
| --- | --- | --- | --- |
| All | hb501 | 5’-AATGATACGGCGACCACCGAGATCTACACTAGATCGCTCGTCGGCAGCGTC | TAGATCGC |
| Cassidy | hb701 | 5’-CAAGCAGAAGACGGCATACGAGATTCAAGTGGTCTCGTGGGCTCGG | CACTTGA |
| WAL | hb702 | 5’-CAAGCAGAAGACGGCATACGAGATATTCCGGGTCTCGTGGGCTCGG | CCGGAAT |
| Hayden | hb703 | 5’-CAAGCAGAAGACGGCATACGAGATCGGTCTAGTCTCGTGGGCTCGG | TAGACCG |
| JT4 | hb704 | 5’-CAAGCAGAAGACGGCATACGAGATGAGATACGTCTCGTGGGCTCGG | GTATCTC |
| Forsling | hb705 | 5’-CAAGCAGAAGACGGCATACGAGATCTATAGCGTCTCGTGGGCTCGG | GCTATAG |
| ARES | hb706 | 5’-CAAGCAGAAGACGGCATACGAGATGACGGAAGTCTCGTGGGCTCGG | TTCCGTC |
| LTER | hb707 | 5’-CAAGCAGAAGACGGCATACGAGATGCACTCTGTCTCGTGGGCTCGG | AGAGTGC |
| AMT1 | hb708 | 5’-CAAGCAGAAGACGGCATACGAGATAAGAACGGTCTCGTGGGCTCGG | CGTTCTT |
| SWP4 | hb709 | 5’-CAAGCAGAAGACGGCATACGAGATCGTCGAAGTCTCGTGGGCTCGG | TTCGACG |
| JD1 | hb710 | 5’-CAAGCAGAAGACGGCATACGAGATTGCTGGTGTCTCGTGGGCTCGG | ACCAGCA |
| Tank 011 | hb711 | 5’-CAAGCAGAAGACGGCATACGAGATACATTCCGTCTCGTGGGCTCGG | GGAATGT |
| EE | hb712 | 5’-CAAGCAGAAGACGGCATACGAGATTCCATCGGTCTCGTGGGCTCGG | CGATGGA |

Supplementary Table 1: Oligos used in Nextera library construction for the wild populations

| Number | Name | Reads (million) | Sequence (Mb) | Coverage |
| --- | --- | --- | --- | --- |
| 1 | Cassidy | 82 | 8209 | 54 |
| 2 | WAL | 30 | 3000 | 19 |
| 3 | Hayden | 62 | 6173 | 41 |
| 4 | JT4 | 45 | 4542 | 30 |
| 5 | Forsling | 49 | 4877 | 32 |
| 6 | Ares | 56 | 5568 | 37 |
| 7 | LTER | 271 | 27108 | 180 |
| 8 | AMT1 | 108 | 10753 | 71 |
| 9 | SWP4 | 111 | 11141 | 74 |
| 10 | JD1 | 49 | 4881 | 32 |
| 11 | Tank011 | 348 | 34836 | 232 |
| 12 | EE | 64 | 6351 | 42 |

Supplementary Table 2: Coverage of sequenced populations.

| Number | Name | Description |
| --- | --- | --- |
| 1 | Date | The date of collection |
| 2 | Males | The total number of males in hydrated samples |
| 3 | Hermaphrodites | The total number of hermaphrodites in hydrated samples |
| 4 | Percent Males | The fraction of observed individuals that were male |
| 5 | Latitude | Latitude of collection site |
| 6 | Longitude | Longitude of collection site |
| 7 | Elevation | Elevation of collection site |
| 8 | Surface area | Surface area of collection site pool |
| 9 | Depth | Depth of collection site pool |
| 10 | Volume | Volume of collection site pool |
| 11 | S/V ratio | Ratio of surface area to volume in collection site pool |
| 12 | pH | pH of tank after hydrating soil |
| 13 | Ap | Average number of alleles per polymorphic allozyme locus |
| 14 | He | Expected number of heterozygotes based on allozyme polymorphism |
| 15 | f | Inbreeding coefficient, based on allozyme data |
| 16 | Fairy shrimp | Presence or absence of unclassified fairy shrimp in hydrated soil |
| 17 | Cladocerans | Presence or absence of Cladocerans in hydrated soil |
| 18 | Leptestheria | Presence or absence of Leptestheria clam shrimp in hydrated soil |
| 19 | Triops longicaudatus | Presence or absence of Triops longicaudatus tadpole shrimp in hydrated soil |
| 20 | Tadpole | Presence or absence of unclassified tadpole shrimp in hydrated soil |
| 21 | Streptocephalus mackeni | Presence or absence of Streptocephalus mackeni fairy shrimp in hydrated soil |
| 22 | Thamnocephalus platyurus | Presence or absence of Thamnocephalus platyurus fairy shrimp in hydrated soil |
| 23 | Eocyzicus | Presence or absence of Eocyzicus clam shrimp in hydrated soil |
| 24 | Tadpole shrimp count | Number of tadpole shrimp observed in hydrated soil |

Supplementary Table 3: A key of abbreviations for the measured environmental variables.

| Name |
| --- |
| Date |
| Percent Males |
| Latitude |
| WAL, Longitude, Thamnocephalus platyurus, Streptocephalus mackeni, Cladocerans |
| Elevation |
| LTER, Surface area, Volume |
| S/V ratio, Depth |
| pH |
| Ap |
| He |
| f |
| Fairy shrimp |
| Leptestheria |
| Triops longicaudatus |
| Forsling, Tadpole |
| Ares, Eocyzicus |
| Tadpole shrimp count |
| Tank011 |
| JD1 |
| SWP4 |
| AMT1 |
| JT4 |
| Hayden |
| Cassidy |

Supplementary Table 4: A key of abbreviations for the measured environmental variables, after combining all redundant (i.e., highly correlated) variables.

| Environmental variable | 25% | 50% | 75% | 90% | 95% | 99% | 99.9% | 99.99% |
| --- | --- | --- | --- | --- | --- | --- | --- | --- |
| % | -13.05 | -13.05 | -13.05 | -13.05 | -13.05 | -13.05 | -13.05 | -13.05 |
| AMT1 | -13.05 | -13.05 | -13.05 | -13.05 | -13.05 | -13.05 | -13.05 | -13.05 |
| Ap | -13.05 | -13.05 | -13.05 | -13.05 | -13.05 | -13.05 | -13.05 | -13.05 |
| Ares | -13.05 | -13.05 | -13.05 | -13.05 | -13.05 | -13.05 | -5.26 | 6.04 |
| Cassidy | -13.05 | -13.05 | -13.05 | -13.05 | -13.05 | -13.05 | -13.05 | -13.05 |
| Cladocerans | -13.05 | -13.05 | -13.05 | -13.05 | -13.05 | -13.05 | -13.05 | -13.05 |
| Date | -13.05 | -13.05 | -13.05 | -13.05 | -13.05 | -13.05 | -13.05 | -13.05 |
| Depth | -13.05 | -13.05 | -13.05 | -13.05 | -13.05 | -13.05 | -13.05 | -13.05 |
| Elevation | -13.05 | -13.05 | -13.05 | -13.05 | -13.05 | -13.05 | -13.05 | -13.05 |
| Eocyzicus | -13.05 | -13.05 | -13.05 | -13.05 | -13.05 | -13.05 | -13.05 | -13.05 |
| f | -13.05 | -13.05 | -13.05 | -13.05 | -13.05 | -13.05 | -0.46 | 12.35 |
| Fairy | -13.05 | -13.05 | -13.05 | -13.05 | -13.05 | -13.05 | -13.05 | -13.05 |
| Forsling | -13.05 | -13.05 | -13.05 | -13.05 | -13.05 | -13.05 | -13.05 | -13.05 |
| Hayden | -13.05 | -13.05 | -13.05 | -13.05 | -13.05 | -13.05 | -13.05 | 6.27 |
| He | -13.05 | -13.05 | -13.05 | -13.05 | -13.05 | -13.05 | -13.05 | 3.38 |
| Hermaphrodites | -13.05 | -13.05 | -13.05 | -13.05 | -13.05 | -13.05 | -1.56 | 8.38 |
| JD1 | -13.05 | -13.05 | -13.05 | -13.05 | -13.05 | 4.05 | 19.68 | 46.94 |
| JT4 | -13.05 | -13.05 | -13.05 | -13.05 | -13.05 | 5.01 | 21.77 | 43.92 |
| Latitude | -13.05 | -13.05 | -13.05 | -13.05 | -13.05 | -13.05 | -13.05 | -13.05 |
| Leptestheria | -13.05 | -13.05 | -13.05 | -13.05 | -13.05 | -13.05 | -13.05 | -13.05 |
| Longitude | -13.05 | -13.05 | -13.05 | -13.05 | -13.05 | -13.05 | -13.05 | -13.05 |
| LTER | -13.05 | -13.05 | -13.05 | -13.05 | -13.05 | -13.05 | -13.05 | -13.05 |
| Males | -13.05 | -13.05 | -13.05 | -13.05 | -13.05 | -5.26 | 7.17 | 20.39 |
| pH | -13.05 | -13.05 | -13.05 | -13.05 | -13.05 | -13.05 | -13.05 | -13.05 |
| S/V | -13.05 | -13.05 | -13.05 | -13.05 | -13.05 | -13.05 | -2.24 | 8.38 |
| Size | -13.05 | -13.05 | -13.05 | -13.05 | -13.05 | -13.05 | -13.05 | -13.05 |
| Streptocephalus | -13.05 | -13.05 | -13.05 | -13.05 | -13.05 | -13.05 | -13.05 | -13.05 |
| Surface area | -13.05 | -13.05 | -13.05 | -13.05 | -13.05 | -13.05 | -13.05 | -13.05 |
| SWP4 | -13.05 | -13.05 | -13.05 | -13.05 | -13.05 | -13.05 | -13.05 | 7.53 |
| Tadpole | -13.05 | -13.05 | -13.05 | -13.05 | -13.05 | -13.05 | -13.05 | -13.05 |
| Tank011 | -13.05 | -13.05 | -13.05 | -13.05 | -13.05 | -13.05 | -13.05 | -7.02 |
| thamnocephalus | -13.05 | -13.05 | -13.05 | -13.05 | -13.05 | -13.05 | -13.05 | -13.05 |
| Triops | -13.05 | -13.05 | -13.05 | -13.05 | -13.05 | -13.05 | -13.05 | -13.05 |
| Volume | -13.05 | -13.05 | -13.05 | -13.05 | -13.05 | -13.05 | -13.05 | -13.05 |
| WAL | -13.05 | -13.05 | -13.05 | -13.05 | -13.05 | -13.05 | -13.05 | -13.05 |

Supplementary Table 5: quantiles of log_10_(Bayes factor) estimates by environmental variable. The percentages at the top are the percentiles that the below Bayes factor quantiles correspond to. The majority of log_10_(Bayes factor) estimates are at the minimum value, -13.05.

| Contig | Range | Env. variable | *X^T^X* Region |
| --- | --- | --- | --- |
| C0001 | 197225:197226 | S/V | None |
| C0001 | 345201:345202 | S/V | None |
| C0001 | 1833709:1833710 | Hayden | R1 |
| C0001 | 2066043:2068856 | SWP4 | R2 |
| C0001 | 2240597:2240598 | f | None |
| C0001 | 3446747:3446748 | f | None |
| C0001 | 4065143:4065185 | SWP4 | None |
| C0001 | 5031843:5031844 | f | None |
| C0001 | 7210188:7210189 | Tank011 | R3 |
| C0001 | 7612097:7613897 | SWP4 | R4 |
| C0001 | 8542258:8542259 | Cassidy | None |
| C0001 | 9538996:9538997 | S/V | None |
| C0001 | 9705793:9706065 | SWP4 | None |
| C0001 | 16028528:16028529 | f | None |
| C0001 | 18294545:18294546 | Hayden | None |
| C0001 | 20549759:20551632 | Tank011 | R7 |
| C0001 | 21876174:21877156 | SWP4 | None |
| C0001 | 24221868:24221869 | f | None |
| C0001 | 26311973:26311974 | f | None |
| C0001 | 27411775:27411776 | f | None |
| C0001 | 27618890:27618891 | Hayden | None |
| C0001 | 30645351:30647994 | Hayden | R8 |
| C0001 | 32119145:32119389 | SWP4 | None |
| C0001 | 32702413:32702414 | S/V | None |
| C0001 | 33839493:33841811 | Hayden | R9 |
| C0001 | 34696220:34696221 | f | None |
| C0001 | 36164194:36164195 | S/V | None |
| C0001 | 37055105:37055106 | f | None |
| C0001 | 40881662:40881663 | f | None |
| C0002 | 3032252:3032253 | S/V | None |
| C0002 | 4630922:4630923 | Tank011 | R11 |
| C0002 | 9464679:9464680 | Ares | None |
| C0002 | 10557700:10558134 | SWP4 | R13 |
| C0002 | 10839998:10840003 | f | None |
| C0002 | 11764243:11764244 | Ares | None |
| C0002 | 12095890:12095891 | He | None |
| C0002 | 14681250:14681251 | S/V | None |
| C0002 | 15740191:15740192 | f | None |
| C0002 | 16322920:16322921 | f | None |
| C0002 | 16656834:16656835 | f | None |
| C0002 | 17201441:17201442 | Hayden | R14 |
| C0003 | 223143:223144 | Ares | None |
| C0003 | 2535351:2535358 | f | None |
| C0003 | 2962792:2962793 | S/V | None |
| C0003 | 3000858:3000891 | S/V | None |
| C0003 | 3242399:3242403 | Hayden | None |
| C0003 | 3763670:3763724 | SWP4 | None |
| C0003 | 4598755:4598756 | Hayden | R15 |
| C0003 | 7468748:7469069 | Hayden | R16 |
| C0003 | 7907524:7907525 | f | None |
| C0003 | 8296179:8296180 | SWP4 | None |
| C0004 | 26995:26996 | f | None |
| C0004 | 499764:499765 | f | None |
| C0004 | 1393362:1393363 | f | None |
| C0004 | 2801686:2801687 | f | None |
| C0004 | 5363921:5363924 | He | None |
| C0004 | 6328985:6328986 | S/V | None |
| C0004 | 7378915:7378916 | f | None |
| C0004 | 8122487:8122778 | SWP4 | None |
| C0004 | 8122605:8122606 | S/V | None |
| C0005 | 64387:64388 | f | None |
| C0005 | 1958135:1958136 | Ares | None |
| C0005 | 3228420:3228421 | Ares | None |
| C0005 | 3298846:3298847 | S/V | None |
| C0006 | 943167:943168 | f | None |
| C0006 | 1841240:1841241 | S/V | None |
| C0007 | 414844:414845 | Ares | None |
| C0007 | 2131561:2131562 | f | None |
| C0007 | 2565501:2565502 | Ares | None |
| C0008 | 225977:225978 | f | None |
| C0008 | 2473479:2473500 | f | None |
| C0009 | 731173:731384 | Ares | None |
| C0009 | 1323327:1323611 | SWP4 | None |
| C0009 | 1452929:1452930 | Ares | None |
| C0011 | 931175:931176 | S/V | None |
| C0012 | 535061:535062 | Ares | None |
| C0015 | 244188:244189 | Ares | None |
| C0025 | 27278:27279 | Ares | R18 |
| C0028 | 32005:32415 | Hayden | R19 |

## *Supplementary Table 6: Regions identified as having at least one site with a Bayes factor over 10^20^. The environmental variable identified as being associated with the region is in the third column, and, if applicable, the region identified as significant by X^T^X is identified in the fourth column. The JT4 and JD1 population dummy variables are excluded from this analysis because their respective populations are closely related, leading to high Bayes factor values across the entire genome.*

## Supplementary figures

Supplementary Figure 1: The minor allele frequency spectrum for all SNPs that passed coverage censoring in the Haplocaller-200ploid polymorphism calling regime, and a projected allele frequency spectrum under neutrality. The 200-ploid setting seems to have prevented most SNPs from being called at all, and those that are called are overwhelmingly low frequency.

Supplementary Figure 2: The minor allele frequency spectrum for all SNPs that passed coverage censoring in the Haplocaller-diploid polymorphism calling regime used throughout the paper, and a projected allele frequency spectrum under neutrality. This set of SNP calls is relatively consistent in the number of high-frequency SNPs called, but low-frequency SNPs are often excluded by the genotyper because of the diploid assumption.

Supplementary Figure 3: The minor allele frequency spectrum for all SNPs that passed coverage censoring in the minimal-assumption samtools polymorphism calling regime, and a projected allele frequency spectrum under neutrality. Like the Haplocaller diploid calls, this set of SNP calls is relatively consistent in the number of high-frequency SNPs called, but low-frequency SNPs are often excluded by the polymorphism caller. There are far more polymorphisms identified here than in the case of the GATK-derived SNP calls, so these calls were not used for fear of false positives.

Supplementary Figure 4: The minor allele frequency spectrum for all SNPs that passed coverage censoring in the UnifiedGenotyper-diploid polymorphism calling regime and a projected allele frequency spectrum under neutrality. Like the Haplocaller diploid calls, this set of SNP calls is relatively consistent in the number of high-frequency SNPs called, but low-frequency SNPs are often excluded by the genotyper because of the diploid assumption.

Supplementary Figure 5: The minor allele frequency spectrum for all SNPs that passed coverage censoring, comparing different SNP calling regimes. Here, allele counts from all populations are combined to give a grand mean. Note the extreme differences in the number of SNPs detected in the samtools and HaplotypeCaller-200ploid approaches.

Supplementary Figure 6: Manhattan plots of single SNP X^T^X values indicating excess differentiation among the 11 populations for regions 1 through 24 as indicated in figure 4. The plots indicate the signal is highly localized, often suggesting a single gene. The red rectangle in each upper plot indicates the region shown in the corresponding lower plot. The “C” and “R” indicators in the titles indicate the contig and region number. “Freq” indicates population-specific allele frequency, and “Cov” indicates normalized, population-specific sequencing coverge.

Supplementary Figure 7: A heat map depicting the absolute correlation coefficients (|r|) between the measured environmental variables, plus, dummy variables indicating each of the 11 populations. Black indicates a high level of correlation, while white indicates a lack of correlation. It is apparent that several of the environmental variables are highly correlated with one another (e.g., Volume and Surface Area) or an environmental variable is highly correlated with a dummy variable (e.g. Surface Area and LTER) indicating that environmental variable is confounded with our sampling of populations.

Supplementary Figure 8: Sites identified as having allele frequencies that vary with an environmental variable. Manhattan plots depicting the log_10_(Bayes factor) for all sites in the genome that were identified by our HMM as having allele frequencies that differ more than expected based on genome-wide population similarity, and that correlate with an environmental variable. Some variables (JT4 and JD1 dummy variables) have clear elevated background that may indicate problems with the statistic when populations are very similar; thus, these region calls may not be reliable.

## Supplementary references

Feder AF, Petrov DA, Bergland AO (2012) LDx: Estimation of Linkage Disequilibrium from High-Throughput Pooled Resequencing Data. *PLoS ONE*, **7**, e48588.

Fu YX (1995) Statistical Properties of Segregating Sites. *Theoretical Population Biology*, **48**, 172–197.

Li H et al. 2009. The Sequence Alignment/Map format and SAMtools. Bioinformatics. 25:2078–2079. doi: [10.1093/bioinformatics/btp352](https://doi.org/10.1093/bioinformatics/btp352).

Li H, Durbin R (2009) Fast and accurate short read alignment with Burrows–Wheeler transform. *Bioinformatics*, **25**, 1754–1760.

Marroni F et al. 2011. Nucleotide diversity and linkage disequilibrium in Populus nigra cinnamyl alcohol dehydrogenase (CAD4) gene. Tree Genetics & Genomes. 7:1011–1023. doi: [10.1007/s11295-011-0391-5](https://doi.org/10.1007/s11295-011-0391-5).

Pirooznia M et al. 2014. Validation and assessment of variant calling pipelines for next-generation sequencing. Hum Genomics. 8:14. doi: [10.1186/1479-7364-8-14](https://doi.org/10.1186/1479-7364-8-14).

Schmucker D, Clemens JC, Shu H *et al.* (2000) Drosophila Dscam Is an Axon Guidance Receptor Exhibiting Extraordinary Molecular Diversity. *Cell*, **101**, 671–684.

Sinsimer KS, Jain RA, Chatterjee S, Gavis ER (2011) A late phase of germ plasm accumulation during Drosophila oogenesis requires Lost and Rumpelstiltskin. *Development*, **138**, 3431–3440.
